# OUTDOOR EXPERIENCES AND OUTDOOR-BASED ACTIVITIES AND INTERVENTIONS FOR INDIVIDUALS WITH SPINAL CORD INJURY: A SYSTEMATIC SCOPING REVIEW

**DOI:** 10.2340/jrm.v57.40705

**Published:** 2025-01-21

**Authors:** Anders O. AABY, Samuel D. WILLIAMSON, Louise S. MADSEN, Thomas MARIBO, Sophie L. RAVN

**Affiliations:** 1 Specialized Hospital for Polio and Accident Victims, Rødovre, Denmark; 2 Department of Psychology, University of Southern Denmark, Denmark; 3 DEFACTUM, Central Denmark Region, Denmark; 4 National Rehabilitation Marselisborg Centre, Denmark; 5 Department of Public Health, Aarhus University, Denmark, Denmark

**Keywords:** nature, outdoor, greenspace, recreation, neurorehabilitation, SCI, spinal cord injury, neurological disorders

## Abstract

**Study design:**

Systematic scoping review.

**Objectives:**

The aim was to identify and synthesize empirical studies exploring outdoor experiences, activities, and interventions in people with spinal cord injury (SCI).

**Methods:**

Systematic searches were performed in 7 bibliometric databases. Unique records were independently screened by 2 authors. Peer-reviewed studies on outdoor experiences, activities, or interventions in adults with SCI were included. This was supplemented by Google Scholar searches and citation tracking. Data from included studies were extracted and analysed in a narrative synthesis.

**Results:**

A total of 89 studies were included. Study findings were catalogued into 9 categories and grouped into 3 themes. Theme 1 covered findings related to the experiences and outcomes of outdoor recreational activities and nature exposure. Theme 2 covered findings on facilitators and barriers related to outdoor recreational activities and nature exposure. Theme 3 covered findings related to outdoor testing of equipment and tools.

**Conclusion:**

People with SCI mainly report positive experiences from engaging with the natural environment and pursuing outdoor activities, but also experienced a range of barriers that need to be considered in both research and clinical practice. Future studies need to explore the effects of outdoor-based rehabilitation, also employing high-quality methods.

## LAY ABSTRACT

This systematic scoping review examined outdoor activities and interventions among people with spinal cord injury. By analysing 89 peer-reviewed studies, the research catalogued findings into 9 categories under 3 themes. The first theme highlighted the varied experiences and outcomes of engaging in outdoor recreational activities and nature exposure. The second theme focused on the facilitators and barriers encountered. The third theme examined outdoor testing of specialized equipment and tools. Participants reported generally positive experiences with outdoor activities and nature, though they faced several obstacles. The findings suggest the need for future research to further investigate outdoor-based rehabilitation and improve methodological quality. Promoting varied outdoor activities could support recovery by providing physical, psychological, and social health benefits. However, successful implementation requires overcoming numerous barriers with strong commitment from health professionals and policymakers. Future studies should explore transitioning indoor rehabilitation to outdoor settings to better understand and enhance health outcomes.

For people living with spinal cord injury (SCI), rehabilitation is crucial for optimizing function across life areas (1, 2). While rehabilitation typically occurs in health facilities, outdoor and greenspace environments can also support people with SCI in everyday life (3). Expanding rehabilitation contexts may encourage people to strive for improvement and learn new strategies beyond the limitations of disability (4).

In recent decades, research linking health and greenspaces has increased and formed an interdisciplinary research field (5–11). The effort to understand the nature-health connection has been aided by the development of Attention Restoration Theory (12) and Stress Reduction Theory (13). Attention Restoration Theory highlights nature’s capacity to restore mental energy depleted by urban environments (12), while Stress Reduction Theory suggests that unthreatening natural environments activates several emotional and physiological stress-reduction responses (13). Indeed, studies have shown benefits of getting away from hospital settings, appreciating nature, and engaging in outdoor activities like kayaking or fishing (3, 14–16).

Despite extensive research, synthesizing findings is challenging due to methodological and contextual diversity. An early review from 2004 explored outdoor experiences of people with SCI but lacked a rigorous approach to searching, screening, and data extraction, relying partly on anecdotal evidence such as personal correspondence and news media stories (3). Later, a 2017 systematic review explored health-promoting nature access for people with mobility impairments, including 3 SCI studies (8) but omitted findings from man-made parks and outdoor community facilities as well as studies on non-health-related outcomes and perspectives on barriers and facilitators. A 2019 systematic review on nature’s psychosocial impact on neurological disability reported no SCI-specific studies, identifying this as an under-researched area (10).

A systematic scoping review is necessary to summarize these diverse findings and guide future research and practice. The aim of this study was therefore to identify and synthesize studies exploring outdoor experiences and outdoor-based activities or interventions among adults living with SCI.

## METHODS

This systematic scoping review follows PRISMA guideline extension for scoping reviews (17), with a pre-registered protocol in the Open Science Framework on 7 April 2022 (available via https://doi.org/10.17605/OSF.IO/5W3ZQ).

### Search strategy

Two search blocks were developed. Block 1 contained outdoor-related terms such as nature, outdoor, outside, greenspace, forest, garden, and more. Block 2 contained terms related to SCI such as spinal cord lesion, spinal trauma, tetraplegia, paraplegia, and more. Search terms were based on the literature and indexes, tested in pilot searches, and used at title/abstract/keyword level or similar. Search terms available as a subject heading were searched as subject headings without explosion and as free text. No database restrictions were used. The literature search was performed as uniformly as possible across databases. The full electronic search string is provided as supplementary information (see Appendix S1).

Final searches were performed in 7 databases, i.e., PubMed, PsycINFO (via OVID), Embase (via OVID), Web of Science, Scopus, Cochrane Library, and CINAHL (added after consultation with a research librarian as not all volumes of Therapeutic Recreation Journal were indexed in the other databases). The main databases were searched on 7 April 2022, and CINAHL on 22 April 2022. Additionally, backward citation tracking and unsystematic searches in PEDro and Google Scholar were conducted using various combinations of the search terms.

### Eligibility criteria

Eligibility criteria were developed prior to screening. Studies were included if they were peer-reviewed; published in English, Danish, Swedish, or Norwegian; based on an adult SCI population (≥ 18 years); and explored or evaluated outdoor experiences or outdoor-based activities or interventions in any form or setting. Studies with mixed samples needed either at least 50% SCI participants or SCI-specific results. Studies merging indoor and outdoor activities were excluded unless outdoor-specific results were reported. No restrictions were imposed on study method or design. Reviews, dissertations, letters, editorials, book chapters, protocols, online registration of interventions, and conference abstracts were excluded as ineligible formats, and studies noted as unavailable were those we could not access, even with the assistance of university research librarians.

### Screening procedure

The search outputs were combined, duplicates removed, and all unique studies were independently screened by 2 authors using Covidence (18). Titles and abstracts were first screened, and non-excludable studies were then full-text screened with exclusion reasons registered for publication purposes. Disagreements were resolved through discussion, with co-authors consulted when necessary. Screening of PEDro and Google Scholar records was conducted by the first author.

### Data extraction and synthesis

Data extracted included author(s), year of publication, country, study setting, outdoor setting and activity, study aim, study design, outcome(s), sample size (N), sample characteristics (age and sex), injury characteristics (injury level, injury completeness, and time since injury), and key findings relevant to this review’s aim. The extracted data were tabulated and are provided as supplementary information (see Appendix S2 for study details and Appendix S3 for main findings). Data extraction was performed independently by 2 authors, and disagreements were resolved by checking the original paper. A third author was consulted when necessary. Two student assistants aided the data extraction process for the descriptive data (i.e., not results). They provided 1 part of the data extraction, while 1 of the authors provided the other part. The first author cross-verified the extractions tables. In 7 cases, corresponding authors were contacted to clarify SCI proportions; 5 of these studies were then included (19–23).

A narrative synthesis was performed to synthesize key findings. This was deemed the most appropriate technique due to methodological heterogeneity in the research field. After familiarization with the data, 1 author initially organized findings into coherent categories and themes, and performed a preliminary narrative synthesis. This was refined by the review team through iterative discussion until consensus was reached.

## RESULTS

### Systematic search results

**Fig. 1:**
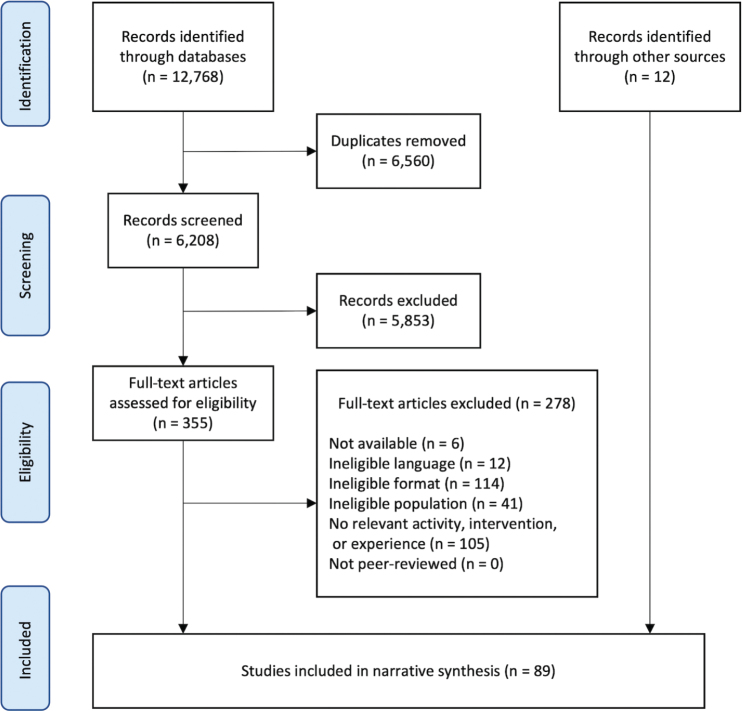
PRISMA flowchart of the systematic search and screening process, including reasons for exclusion. Final search was performed on 7 April 2022.

The systematic searches identified 6,208 unique records. Of these, 5,853 were excluded in title and abstract screening, and 278 were excluded in full-text screening, leaving 77 eligible studies. With 12 additional eligible studies identified through other sources (i.e., backward citation tracking and Google Scholar), a total of 89 studies were included in this review (see PRISMA Flowchart in Fig. 1).

### Study characteristics of the included studies

Most studies were conducted in North America (*n* = 62), followed by Western Europe (*n* = 14), and Australia (*n* = 4). Sample sizes ranged from case studies (*n* = 1) to large surveys (*n* = 920 with approximately half having fewer than 20 participants. In terms of sex, 94% included more than 50% men, while 77% included more than two-thirds male participants. Mean age was not reported consistently, but for the 68 studies that did, the mean age ranged from 24.5 years to 64 years. There was considerable variation in how injury characteristics were reported, so a summary is not pertinent. Most studies were quantitative (*n* = 45), followed by qualitative (*n* = 36), and mixed-methods (*n* = 8). A complete list of all the extracted study characteristics is provided as supplementary information (see Appendix S2).

### Narrative synthesis

**Fig. 2:**
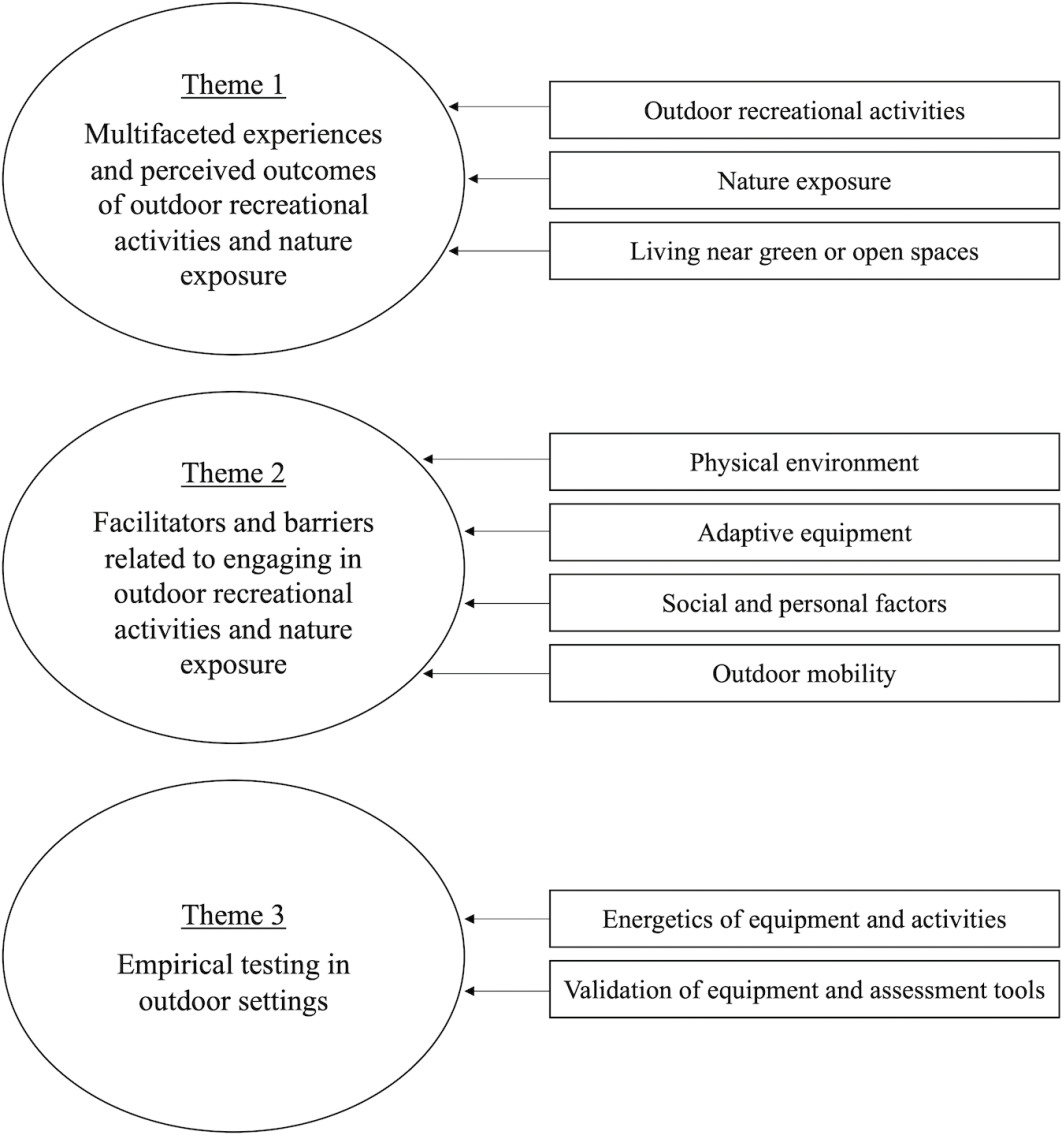
Overview of the categorization of findings in the narrative synthesis. Nine meaningful categories were developed, which were grouped into 3 overarching themes.

The 89 included studies explored a wide range of outdoor contexts and activities, employed various methodological approaches, and focused on different aspects or effects of outdoor experiences. Findings were organized into 9 categories and grouped into 3 overarching themes. For a visual overview of the categorization, see Fig. 2.

This categorization was based on findings, so many of the included studies are represented across multiple themes and categories. The 3 themes and the 9 categories are described below. A complete list of the extracted main findings is provided as supplementary information (see Appendix S3).

*Multifaceted experiences and perceived outcomes of outdoor recreational activities and nature exposure (theme 1).* This theme synthesizes positive and negative experiences of engaging in outdoor recreational activities and nature exposure. The first category, “outdoor recreational activities”, covers experiences in outdoor recreational activities such as hiking, skiing, gardening, kayaking, and more. The second category, “nature exposure”, includes experiences of walking/wheeling or simply being outdoors and in nature. The third category, “living near green and open spaces”, covers health outcomes related to living close to green and open spaces.

*Outdoor recreational activities.* This category was the most prominent with 30 studies (14–16, 20, 21, 24–48). This included degree of participation, general experiences, perceived physical and psychosocial benefits, and negative experiences.

People with SCI were generally interested in participating in outdoor recreational activities (24), and 1 study found that 26% set outdoor-related rehabilitation goals (25). The most popular outdoor recreational activities were wheeling, hand-cycling, kayaking, and sit-skiing (24), but the included studies showed that people with SCI engaged many other activities like hunting and fishing (14, 15, 26, 27), scuba diving (28), horseback riding (24), sailing (29, 30), and more. One study showed an 86% decrease in outdoor activities post-SCI (31), and 2 studies showed reduced or lack of participation in outdoor recreational activities (16, 21).

In terms of effects of participation in outdoor recreational activities, 1 intervention study showed general satisfaction as well as a decrease in barriers and increased positive affect and self-efficacy among participants in a 3-day cottage programme compared with a non-randomized control group (16). The differences in self-esteem and negative affect were not significant (16).

Across studies exploring the experience of different outdoor recreational activities, participants expressed joy, pleasure, well-being, or enthusiasm when they engaged in skiing (20,32), water-skiing (33), kayaking (21, 34–36), sailing (29,30), hunting and fishing (26, 27), hiking (37, 38), and hand-cycling (21, 32, 33, 35). Participants also regularly experienced a sense of achievement, self-esteem, or competence (20, 29, 34, 39). Participation in outdoor recreational activities was also closely related to recovery, re-integration, and identity reconstruction (24, 26, 40, 41). In addition to these positive experiences, 1 study noted that people with SCI were frustrated with the patronizing comments they received (24). Findings from some studies (20, 21, 29, 30, 37, 39) were derived from evaluations of specific programmes, while the remaining findings reflect the experiences of individuals with SCI engaging in these activities within their everyday lives.

Focusing first on land-based activities, 2 studies evaluating skiing programmes (20, 39) and 1 study exploring experiences with prior use of sit-skiing (42) showed that participants experienced increased quality of life (42), improved physical condition, self-worth, and satisfaction with their body (39), in addition to feeling less anxious, more prepared, and empowered (20). While participants in 1 study found skiing to be daunting (20), another study reported that participants felt safe (42). Like skiing, hand-cycling made participants nervous, and some were hesitant to try it out in a study describing a 10-session recreational programme (21). However, with encouragement all participants tried it and found it enjoyable (21). Another study found hand-cycling to be the most popular activity on offer (43). In other studies where participants reflected on their use of hand-cycling, it was described as providing a sense of freedom and independence (33, 41). Furthermore, people with SCI preferred biking outside (44), and it was viewed as a way of getting back into the woods (32). Getting back into nature was also appreciated during a skiing programme (20) and a hiking programme (37), as well as when reflecting on previous hiking experiences (41).

Gardening was one of the activities in which people with SCI were most likely to experience changed performance when reflecting on leisure activities (27), and only around 8% had engaged in gardening in the previous 3 days (46). In reflecting on leisure activities, gardening was found in 1 study to be therapeutic and relaxing (28). Similar to gardening, when reflecting on hunting and fishing, people with SCI described having challenges in performance or at least in getting the full experience (45), but also as a way to relax (26), a diversion from worry (47), and a source of well-being (27).

In terms of water-based activities, similar sentiments of appreciating the natural scenery and the calming atmosphere on the water were expressed when reflecting on water skiing and kayaking (34, 36, 43), as well as during sailing programmes (29, 30). In 3 studies, participants reflected on their participation in sea kayaking expeditions provided by an outdoor experience organization and described it as a means of immersing oneself in the natural world (34, 36, 48). Participants in 1 study expressed feeling safe during kayaking (36), while they expressed fears of capsizing and bad weather in another study (34). This was the only negative experience reported. Kayaking was described as an equalizer because participants felt on the same level as their able-bodied peers (34, 36, 48). It further provided a sense of freedom, supported their adjustment process and mental health, and offered physical health benefits like strength, stamina, and balance (34, 36, 48). A sense of freedom was also described when reflecting on swimming and during sailing programmes (24, 29, 30). Participants also experienced that independent sailing challenged stigmas concerning their abilities (29).

*Nature exposure.* The appreciation of being outdoors and in the natural environment was not just expressed during outdoor recreational activities. A total of 13 studies were categorized as reporting findings on the experience of nature exposure while simply walking/wheeling outside or just being in nature (14, 15, 27, 33, 49–57). For instance, some expressed a newfound appreciation for nature following their injury (33, 54). Furthermore, participants trying out an exoskeleton outside commented on the positive effects of feeling the sun and kicking leaves (53). Simply getting outside was described as a much appreciated escape from the hospital setting (14) and from feeling caged (15). Lastly, outdoor activities (i.e., walking/wheeling, camping, and being in nature) were among the 6 most likely activities (out of 21 activities) for people with SCI to be interested in and to endorse as a source of well-being (27).

In addition to generally positive experiences, some adverse effects and negative experiences associated with nature exposure were expressed. A few studies noted that outdoor wheeling was associated with increased pain (55–57). Outdoor wheeling and being in the garden or on the street were all found to be associated with a higher risk of falling (50–52). This led to worry and vigilance when scanning the environment, which reduced their enjoyment of being outside (49).

*Living near green or open spaces.* Four studies investigated whether living near greenspaces and open spaces was related to greater participation, health, functioning, and well-being for people with SCI (58–61). Collectively, they showed inconsistent results. First, living in areas with a large proportion of open spaces approximately doubled the odds of reporting full participation (61) and had approximately half the odds of reporting poor health (58). Furthermore, proportion of park space was associated with higher functioning (59). Conversely, living in areas with moderate amounts of greenspace and open spaces was associated with greater depression and less positive affect compared with living in areas with less greenspace and open spaces (60).

*Facilitators and barriers related to engaging in outdoor recreational activities and nature exposure (theme 2).* In theme 2, factors that either promote or hinder participation in outdoor recreational activities and nature exposure were identified and grouped. It consisted of 4 categories: physical environment, adaptive equipment, social and personal factors, and outdoor mobility.

*Physical environment.* This category included 18 studies (15, 19, 22, 35, 41, 62–74). These included lack of or steep kerb cuts; lack of or poorly maintained sidewalks; uneven, steep, soft, wet, or slippery terrain; grass, mud, sand, stones and gravel; and stairs (15, 19, 35, 41, 63–69). Furthermore, climate and weather were generally reported as barriers to participation (64, 70). Winter conditions, specifically, were found to be a barrier due to slippery roads, increased pain, and discomfort (63, 68, 69, 71–74). Conversely, summer conditions were both a barrier and a facilitator (68, 69). In addition to seasonal effects, rain was noted in 3 studies as being an issue with mobility assistive devices (15, 66, 67).

*Adaptive equipment.* In total, 18 studies were categorized here (14, 20, 24, 29, 32, 37, 48, 75–85). First, it was highlighted as being of importance to be able to pursue outdoor recreational activities (24, 75), and specifically in terms of skiing (20), fishing (14, 32), kayaking (48), sailing (29), and hiking (37). Three studies also highlighted high cost (24, 32), poor condition (29), and reliance on other people’s assistance (24).

A range of studies showed the mitigating effects of having mobility assistive devices that could traverse difficult terrain (32, 37, 76–79). Specifically in terms of exoskeletons, the most common location of usage was outside (81), and they enabled walking on various outdoor terrain (82), increased outdoor walking speed (83, 84), and significantly improved outdoor 6-min walking test (85).

*Social and personal factors*. A total of 10 studies were included in this category (20, 21, 24, 28, 29, 37, 40, 41, 75, 86). First, social encouragement was a facilitator of outdoor recreation (21, 24). Conversely, needing assistance, lack of volunteers, health concerns, transportation, and scheduling were highlighted as barriers to outdoor recreation across studies (24, 28, 29, 37, 41). A personal barrier to pursuing outdoor recreational activities was lack of knowledge on possibilities, different kinds of adaptive equipment, and how to use them (21, 75). Conversely, having someone to consult was described as a vital social facilitator (75).

Volunteers were instrumental in a hiking excursion programme, but, on a personal level, relinquishing control and accepting dependence on volunteers was difficult for some participants (37). Acceptance, in the sense of being able to let go of the past self in developing a new identity, was found to be associated with engagement in new outdoor activities (40). Similarly, constructing the SCI in a “quest narrative” facilitated engagement in sports and outdoor recreation compared with the “restoration narrative” (86).

*Outdoor mobility.* Outdoor mobility is a facilitator or barrier to engaging in outdoor activities, and 4 of the included studies explored outdoor mobility (87–90). One study was able to improve walking ability in a 4-week training programme, but improvements were not maintained at follow-up (87). Another study showed that a walking speed of 0.59 m/s was able to distinguish between people who were able to walk outdoors and those who were not (88). Lastly, motor strength, light touch sensation, and lower extremity motor scores were key predictors of outdoor mobility (89, 90).

*Empirical testing in outdoor settings (theme 3).* This theme included studies that used an outdoor setting to perform empirical testing and validation of equipment and assessment tools. In the first category, studies that explored energetics (i.e., energy use) of equipment or activities were synthesized. In the second category, studies that tested and validated non-wheelchair or exoskeleton equipment in an outdoor setting or any other assessment tools related to the outdoor context were synthesized.

*Energetics of equipment and activities.* Ten studies were categorized as reporting findings related to energetics (91–100). These studies showed participants using less physiological effort when wheeling than when walking outside (92), when using ultralight wheelchairs (93), when using 2-arm chairs compared with 1-arm chairs (94), when using power-assisted compared with manual wheelchairs (95), and when using a mobility assistance dog (98). Further, propulsion forces were significantly higher when wheelchairs are propelled over grass, pavement, and uphill (96), as well as across aggregated outdoor terrain compared with smooth terrain (97). Two studies used heart rate to indicate energy use during outdoor tasks and found it was a reliable indicator when propelling a wheelchair outdoors (99), but could not reliably predict oxygen uptake (100).

*Validation of equipment and assessment tools.* Five studies were categorized as testing and validating equipment or assessment tools related to outdoor contexts (23, 101–104). Three studies have developed and validated equipment in outdoor setting: a fatigue meter (101), a device to quantify physical activity using wheel pushes (102), and a set of crutches to assess load on upper extremities (23). In terms of assessment tools, the outdoor 6-min walking test was more representative of actual performance as the indoor version tended to underestimate walking ability (103). Lastly, the outdoor domain in a physical activity scale showed poor reliability (104).

## DISCUSSION

### Summary of findings

Overall, studies indicate frequent participation in outdoor recreational activities like wheeling, hand-cycling, kayaking, and sit-skiing among people with SCI, offering positive experiences such as joy, self-efficacy, and freedom. Being outdoors helped escape hospital settings and appreciate nature, although negative experiences like patronization, safety concerns, and increased pain were noted. Proximity to greenspaces correlated with better physical health and activity levels but also with depression and reduced positive affect.

For individuals with SCI, barriers to outdoor activities and nature exposure included inaccessible environments, adverse weather, high costs, and lack of adaptive equipment. Knowledge of adaptive equipment and support from volunteers and family were crucial facilitators, whereas needs for assistance, insufficient volunteer availability, health issues, and transport logistics posed significant challenges.

Research showed that outdoor equipment demonstrated distinct differences in energetics and propulsion forces across various activities. Tools like fatigue meters and wheel push counters were effectively validated outdoors. The 6-min walking test, administered in a community setting, provided a more accurate performance representation than when conducted indoors.

### Study findings in context

Positive experiences in outdoor recreation like joy, freedom, and recovery align with the 2004 review on SCI and outdoor experiences (3). This review adds empirical weight, highlighting nuances related to positive and negative experiences of outdoor activities, nature exposure, and living near greenspaces. Barriers like transportation, social support, and knowledge noted in the 2004 review (3) are further detailed here to include the physical environment, weather, cost and availability of adaptive equipment, and social factors like health concerns and acceptance. Additionally, this review uniquely emphasizes the physical requirements like walking speed and motor strength as well as specialized equipment, such as ultralight and power-assisted wheelchairs and mobility assistance dogs, which enhance accessibility and reduce strain, fostering independence. Outdoor-specific assessments, like the 6-minute walking test, were validated for measuring functional capacity in natural settings.

Findings from related disability research reflect similar positive outcomes in outdoor activities such as kayaking and sailing (105), paddling (106), skiing (107), and hiking (108), alongside a sense of freedom and relaxation in nature (109). Increased mood and well-being, relaxation, sense of competence, self-esteem, physical strength, and social connectedness were also noted in 3 recent reviews focusing on engagement with nature for people with physical disabilities (8), brain injury (9), and neurological disability (10).

However, there were also notable differences. Cognitive benefits from nature engagement were observed in other reviews (8–10) but not found here, possibly due to the focus on different populations like those with brain injuries (9) or neurological disabilities (10). Additionally, while nature engagement often provided pain relief for those with physical disabilities (110, 111), it frequently resulted in increased pain for individuals with SCI, likely because their pain arose from active participation in outdoor activities such as wheeling.

The findings align with Attention Restoration Theory (12) and Stress Reduction Theory (13), suggesting nature’s role in restoring attention and reducing stress, illustrated through activities like sea kayaking. Other studies within the SCI field have shown a stress-reducing response from visual stimulation with indoor bonsai trees (112) and increased mood and relaxation using virtual reality to view natural scenery (113). Furthermore, living near greenspaces correlates with health and participation outcomes, but also with depression and less positive affect (58–61). This could be because the areas are not accessible. This is underscored by research suggesting that people with disabilities feel a sense of exclusion and outsideness when greenspaces are not accessible (114), highlighting the importance of accessibility in greenspace design.

This review also suggests that recreational activities themselves offer physical, psychological, and social health benefits. However, the outdoor setting could still enhance these effects compared with an indoor environment, as research suggests the natural environment encourages physical activity, improves mental health, and facilitates social cohesion (115–117).

### Clinical implications

Given the positive experiences and noted barriers for individuals with SCI engaging in outdoor activities, this review underscores that rehabilitation should not solely occur within the confines of indoor facilities and points to several crucial implications for clinical practice, rehabilitation, and policy formulation. Integrating outdoor recreational activities such as kayaking, hand-cycling, and gardening into routine rehabilitation programmes could enhance physical, psychological, and social well-being. As such, there is a clear need for training healthcare providers on the benefits and logistics of implementing outdoor-based interventions. This includes understanding the specific needs of people with SCI and how to safely incorporate outdoor activities into therapeutic practices. Furthermore, addressing physical, environmental, technical, personal, and social barriers is essential for successful outdoor rehabilitation. Clinics and rehabilitation centres should work with local governments and organizations to improve accessibility of parks, trails, and outdoor recreational facilities. This includes ensuring accessible transport and the availability of adaptive equipment. There is a need for research institutions, manufacturers, and community organizations to continue the development and distribution of cost-effective and sturdy adaptive equipment that meets the practical needs of individuals with SCI and enhances independence in outdoor settings.

The findings on outdoor mobility offer essential insights into the physical requirements for independent movement outdoors, including walking speed and motor strength, which are predictive of outdoor ambulation abilities in people with SCI. For example, a minimum walking speed of 0.59 m/s was shown to differentiate those who can walk outdoors from those who cannot, while motor strength, light touch sensation, and lower extremity scores further predict outdoor mobility, suggesting that rehabilitation professionals should incorporate assessments of these physiological indicators to tailor interventions effectively and to set realistic mobility goals that facilitate outdoor engagement.

This review also highlights the importance of equipment validation for outdoor use. Studies on energy expenditure and propulsion forces indicate that using aids like ultralight wheelchairs, power-assisted devices, and mobility assistance dogs can reduce physical strain during outdoor activities. Additionally, a community 6-min walking test offers a more accurate assessment of actual outdoor mobility compared with indoor tests, which tend to underestimate capacity in real-world settings. These validated tools should be integrated into clinical practice to ensure that the functional abilities of individuals with SCI are accurately represented and supported in outdoor contexts.

In terms of social barriers, building strong support networks of peers, friends, family, and volunteers could help facilitate participation in outdoor activities. Programmes designed to increase social interactions and community bonding through group activities could be especially beneficial. As an overarching theme, policymakers should be informed of the benefits of outdoor activities for people with SCI. This could facilitate and promote the development of policies that support and fund accessible greenspaces and outdoor recreational areas.

Of note, the present study found only a few intervention studies and no studies that applied more rigorous methods to test the effect of outdoor-based interventions. This lack of strong intervention studies lessens the certainty of conclusions that can be drawn to guide clinical practice. It remains unclear how changes in rehabilitation setting specifically affect health outcomes. This uncertainty is important to address in future research. Furthermore, we found no studies that explored the conversion of traditionally indoor rehabilitation activities (e.g., transfer training) to outdoor settings, thereby also providing no strong evidence base for clinical practice.

### Limitations

The present study holds several strengths, like a broad and rigorous search strategy, but also limitations. First, we applied a broad search method to map studies of outdoor experiences, activities, and interventions in SCI. However, this still only identifies studies that mention the search words in the title, abstract, or keywords, or were indexed accordingly. This meant that there are likely studies that have a relevant outdoor activity that have not appeared in our searches. We have attempted to address this issue by conducting thorough searches using Google Scholar, but there are probably still records not identified. This could be in participation research, but also specific outdoor activities that we did not include in our search. This could also be true of studies using mixed study samples (e.g., mixed neurological disorders) or specific SCI samples (e.g., spina bifida), which were not included in the search terms.

Furthermore, the literature search was concluded over 2 years prior to publication, resulting in the exclusion of potentially relevant studies published after April 2022. Given the current pace of research publication, it would be advisable to conduct an updated systematic review within the next 3–5 years to ensure the inclusion of the most recent evidence.

Another limitation involves the language restrictions applied. We included studies published in English, Danish, Swedish, and Norwegian but excluded 6 records based on language. Although it is unknown whether these studies would have met our eligibility criteria, this remains a potential limitation.

A further limitation is related to the considerable variation in the studies. SCIs are highly different in terms of consequences for physical abilities, both due to differences in level and completion of injury, but also due to the large variations in associated symptoms such as pain, spasticity, or gut issues (118). In this review, we have not grouped findings based on injury characteristics or associated symptoms. Such characteristics could potentially serve as an important difference between the studies and related findings in addition to being an important factor in clinical decision-making.

Another limitation is the deviation from the protocol regarding the assessment of risk of bias for the studies included. We chose to ignore this due to the large number and diversity of the studies in the current study. This decision after the fact means that the quality of the studies and the associated risks of bias were not evaluated systematically.

### Future directions

It is crucial to advance our understanding of the specific role that the outdoor natural environment plays compared with the recreational activity. Future studies need to develop and employ rigorous methods and measurement scales to begin teasing out these effects. Furthermore, most research has focused on experiences and perceived benefits of physical activities, so studies need to investigate outdoor pursuits such as horticultural therapy (119), forest bathing (120), and green care farming (121). Similarly, future studies need to explore the experiences and effects of transforming traditional indoor rehabilitation practice to an outdoor context. There could potentially be a set of novel barriers and facilitators associated with this move. Lastly, most research has been conducted in North America, which has a strong culture of outdoor recreational pursuits facilitated by private organizations and volunteers. In the Scandinavian countries, this is much less pronounced as rehabilitation is largely organized within the public health system. Additionally, policy contexts differ by region, with some countries enforcing legal frameworks that secure access to public spaces like parks and forests for people with disabilities. This highlights the importance of examining how national policies and public health infrastructures shape the strategies for outdoor rehabilitation. Related disability research indicated that Scandinavian health professionals express a lack of experience and knowledge necessary to design and implement outdoor rehabilitation programmes (4), underscoring the need for research into interdisciplinary training and knowledge-sharing to support high-quality, outdoor-based rehabilitation services.

## Supplementary Material

OUTDOOR EXPERIENCES AND OUTDOOR-BASED ACTIVITIES AND INTERVENTIONS FOR INDIVIDUALS WITH SPINAL CORD INJURY: A SYSTEMATIC SCOPING REVIEW
