# Supplementary material for: OUTDOOR EXPERIENCES AND OUTDOOR-BASED ACTIVITIES AND INTERVENTIONS FOR INDIVIDUALS WITH SPINAL CORD INJURY: A SYSTEMATIC SCOPING REVIEW
Source: J Rehabil Med. 2025 Jan 21;57:40705. doi: 10.2340/jrm.v57.40705 (PMC11770228; doi:10.2340/jrm.v57.40705)
Supplement: OUTDOOR EXPERIENCES AND OUTDOOR-BASED ACTIVITIES AND INTERVENTIONS FOR INDIVIDUALS WITH SPINAL CORD INJURY: A SYSTEMATIC SCOPING REVIEW [file JRM-57-40705-s1.pdf]

Supplementary Information

Search #	Search terms
1	Urban area [SH]
2	Rural area [SH]
3	Forest [SH]
4	Wilderness [SH]
5	Landscape [SH]
6	Horticulture [SH]
7	Nature*
8	Outdoor*
9	Outside*
10	”Greenspace*”
11	”Green space*”
12	”Bluespace*”
13	”Blue space*”
14	”Greyspace*”
15	”Grey space*”
16	”Open space*”
17	Forest*
18	Garden*
19	Wilderness*
20	eco* NOT econ* NOT ecol* NOT ecos*
21	Biophilia*
22	Adventur*
23	Countryside*
24	Landscape*
25	Botan*
26	Horticultur*
27	#1 OR #2 OR #3 OR #4 OR #5 OR #6 OR #7 OR #8 OR #9 OR #10 OR #11 OR #12 OR #13 OR #14 OR #15 OR #16 OR #17 OR #18 OR #19 OR #20 OR #21 OR #22 OR #23 OR #24 OR #25 OR #26
28	Spinal cord injury [SH]
29	Spinal cord lesion [SH]
30	Spine injury [SH]
31	Quadriplegia [SH]
32	Paraplegia [SH]
33	Spastic paraplegia [SH]
34	“Spinal cord injur*”
35	“Spinal cord lesion*”
36	“Spinal cord trauma*”
37	“Spinal injur*”
38	“Spinal lesion*”

39	“Spinal trauma*”
40	Tetraplegi*
41	Paraplegi*
42	Quadriplegi*
43	#28 OR #29 OR #30 OR #31 OR #32 OR #33 OR #34 OR #35 OR #36 OR #37 OR #38 OR #39 OR #40 OR #41 OR #42
44	#27 AND #43

Notes.
Searched in “title, abstract, heading word, drug trade name, original title, device manufacturer, drug manufacturer, device trade name, keyword heading word, floating subheading word, candidate term word”.
[SH] = Subject Heading

Supplementary information

Appendix 2. Study characteristics of the included studies.

Author (year)	Country and study setting	Sample Size (n)	Sample Characteristics	Injury Characteristics	Study Aim	Outdoor setting and activity	Study Design	Study Outcome(s)
Alsaleem et al. (2019)	USA, Community sample	n = 8*	Men: 63%<br>Women: 37%<br><br>Mean age: 43.4 years (range 20-67)	Tetraplegia: 7<br>Not specified: 1<br><br>No data on completeness.<br><br>Time since injury not reported.	To explore the usability and experiences of Tetra-Ski and the shared control system.	An outdoor skiing session with a trainer at a ski resort.	Mixed methods, pre-post design	The Psychosocial Impact of Assistive Devices Scale (PIADS)<br><br>Single items to measure preparedness and anxiety<br><br>Experiences and feedback
Balbale et al. (2017)	USA, Community sample	n = 9	Men: 88.9%<br>Women: 11.1%<br><br>Mean age: 64 years (range: 36-86)	Paraplegia: 75%<br>Tetraplegia: 25%<br><br>No data on completeness.<br><br>Time since injury: 21.8 years (range: 2-59)	To explore perceptions and experiences related to participation.	Participants described different activities they participated in; some of which were outdoors.	Qualitative, photovoice design	N/A
Barbin et al. (2008)	France, Community sample	n = 10	Men: 70%<br>Women: 30%<br><br>Mean age: 32.1 years (range: 22-40)	Level of injury: C7 = 3, T4 = 2, T10 = 2, T12 = 1, L1 = 2<br><br>No data on completeness.<br><br>Time since injury: 5.1 years (range 1-10)	To explore the impact of a 1-week skiing program.	5 days, 5 h/day adapted skiing program accomplished with the assistance of a personal physical educator.	Quantitative, pre-post design	Physical Self Inventory (PSI-6)<br><br>Physical Self Perception Profile (short version)
Barclay et al. (2016)	Australia, Community sample	n = 17	Men: 70.6%<br>Women: 29.4%<br><br>Mean age not reported.<br>Age range: 18-85 years	Incomplete tetraplegia: 4<br>Complete tetraplegia: 13<br><br>Time since injury: 14 years (range: 4-29 years).	To explore factors related to social and community participation.	Participants described participating in different outdoor activities.	Qualitative, semi-structured interview design	N/A
Beekman et al. (1999)	USA, Clinical sample	n = 74	Men: 93.2%<br>Women: 6.8%<br><br>Mean age: 26.2 years (range: 17-50)	Complete tetraplegia: 30<br>Complete paraplegia: 44<br><br>Time since injury: 5.1 months.	To compare wheelchair propulsion in ultralight and standard wheelchairs.	Propelling around an outdoor track.	Quantitative, quasi-experimental design	Speed, distance traveled and oxygen use.
Berliner et al. (2021)	USA, Clinical sample	n = 129	Men: 83.6%<br>Women: 16.4%<br><br>Mean age not reported for whole sample.	Cervical: 129<br><br>Time since injury not reported.	To explore predictors of outdoor walking ability.	Participants self-reported outdoor walking ability.	Quantitative, retrospective cohort design	Outdoor walking ability (self-reported).

Block et al. (2005)	USA, Community sample	n = 33*	Not reported	Not reported	To describe the Shake-It-Up program	Shake-It-Up is a ten-session program with seminars and recreational group activities, including five outdoor sessions with kayaking, sailing, fishing, kite-flying, and hand-cycling).	Qualitative, descriptive study design	N/A
Boschen et al. (2005)	Canada, Community sample	n = 100	Men: 75.0%<br>Women: 25.0%<br><br>Mean age: 39.95 years (SD: 13.63)	Paraplegia: 42%<br>Tetraplegia: 58%<br><br>Complete: 37%<br>Incomplete: 63%<br><br>Time since injury not reported.	To compare community integration and quality of life of people with SCI with their support providers and non-SCI.	Participants described different barriers to participation; some of which were related to outdoors.	Quantitative, cross-sectional case-control design	The Re-Integration to Normal Living Index (RNL)<br><br>Quality of Life (QOL)<br><br>Locus of Control (LOC)<br><br>Measure of the Quality of the Environment (MQE)
Botticello et al. (2014)	USA, Community sample	n = 508	Men: 80.5%<br>Women: 19.5%<br><br>Mean age: 44 years (SD: 16.6).	Paraplegia: 48.6%<br>Tetraplegia: 51.4%<br><br>No data on completeness.<br><br>Percentage injured within previous 2 years: 37.0%	To explore the associations between built environment and community participation.	The proportion of open space was specified by summing all the natural land use and land cover including undeveloped forest and wetlands, cultivated farmland, and beach or waterfront.	Quantitative, cross-sectional survey design	Craig Handicap Assessment and Reporting Technique (CHART)
Botticello et al. (2015)	USA, Community sample	n = 503	Men: 80.5%<br>Women: 19.5%<br><br>Mean age: 44.5 years (range: 18-89)	Paraplegia: 48.7%<br>Tetraplegia: 51.3%<br><br>Complete: 57.8%<br>Incomplete: 42.8%<br><br>Percentage injured within previous 2 years: 37.2%	To explore the associations between built environment and perceived health.	The proportion of open space was specified by summing all the natural land use and land cover including undeveloped forest and wetlands, cultivated farmland, and beach or waterfront.	Quantitative, cross-sectional survey design	Single item on perceived health from the SF-36
Botticello et al. (2019)	USA, Community sample	n = 402	Men: 78.1%<br>Women: 21.9%<br><br>Age groups:<br>18-35 years = 29.9%<br>36-50 years = 35.3%<br>> 50 years = 34.8%	Paraplegia: 47%<br>Tetraplegia: 53%<br><br>Complete: 42.3%<br>Incomplete: 57.7%<br><br>Percentage injured 3 years or more: 49.8%	To explore the associations between built environment and physical functioning.	Survey data geocoded to represent land use, park space, residential density, and destination density.	Quantitative, cross-sectional survey design	Four domains of the Spinal Cord Injury-Functional Index/Capacity (SCI-FI/C): basic mobility, wheelchair mobility, self-care, and fine motor function.
Butler et al. (2008)	USA, Community sample	n = 45	Men: 100%<br><br>Mean age: 54.2 years (SD: 16.6)	Paraplegia: 24<br>Tetraplegia: 21<br><br>Complete: 16<br>Incomplete: 29<br><br>Time since injury: 18.0 years (SD: 16.6)	To test the reliability and validity of a SCI physical activity instrument.	One of the sub-domains was outdoor work/gardening.	Quantitative, cross-sectional scale validation	The Physical Activity Scale for Individuals with Physical Disabilities (PASIPD).

Caldwell et al. (1994)	USA, Clinical sample	n = 20	Men: 75%<br>Women: 25%<br><br>Mean age: 30.2 years	Paraplegia: 35%<br>Tetraplegia: 65%<br><br>No data on completeness.<br><br>Time since injury not reported.	To explore perceptions of therapeutic recreation.	Participants were interviewed about their experiences of therapeutic recreation; some of which were outdoors.	Qualitative, semi-structured interview design	N/A
Caro et al. (2020)	Brazil, Community sample	n = 11	Men: 72.7%<br>Women: 27.3%<br><br>Mean age: 38.3 years (range: 23-61).	Cervical: 18.2%<br>Thoracic: 72.7%<br>Lumbar: 9.1%<br><br>Complete: 9.1%<br>Incomplete: 90.9%<br><br>Time since injury: 12.8 years.	To explore factors related to functional mobility with a wheelchair.	Barriers related to the outdoor environment.	Quantitative, cross-sectional descriptive survey design	The Person Identification with Spinal Cord Injury Form.
Carpenter (1994)	Canada, Community sample	n = 10	Men: 90%<br>Women: 10%<br><br>Mean age: 31.8 years (range: 21-39)	Cervical: 60%<br>Thoracic: 40%<br><br>No data on completeness.<br><br>Time since injury: 4.3 years (range: 3-5)	To explore how persons conceptualize the experience of spinal cord injury.	Participants described their experiences of living with a spinal cord injury. Some were related to the outdoor environment.	Qualitative, semi-structured interview design	N/A
Carver et al. (2016)	USA, Community sample	n = 61*	Not reported	Not reported	To explore the perspectives that assistive technology device users have toward their devices	Participants described different barriers to navigating the outdoor environment.	Mixed methods cross-sectional survey and semi-structured interview design	Functional mobility assessment (FMA).
Casey et al. (2009)	Ireland, Community sample	n = 6	Men: 50%<br>Women: 50%<br><br>Mean age not reported.	Paraplegia: 66.7%<br>Tetraplegia: 33.3%<br><br>No data on completeness.<br><br>Time since injury not reported.	To explore the meaning of kayaking for persons with spinal cord injury.	Participants were regular sea kayakers and reflected on their experiences pursuing this activity.	Qualitative, Unstructured ethnographic interview design	N/A
Cerny et al. (1980)	USA, Community sample	n = 11	Men: 90.9%<br>Women: 9.1%<br><br>Mean age: 26.8 years (range: 19-42)	Thoracic: 54.5%<br>Lumbar: 45.5%<br><br>No data on completeness.<br><br>Time since injury not reported.	To compare energy requirements of wheelchair propulsion and walking.	Participants walked and propelled their wheelchair around a 60.5m level outdoor cement track.	Quantitative, comparative study design	Velocity, stride length, heart rate, oxygen consumption, and respiratory quotient.

Champagne et al. (2016)	Canada, Community sample	n = 13	Men: 76.9%<br>Women: 23.1%<br><br>Mean age: 40.4 years (SD: 10.3)	AIS neurological level was between C5 and T11.<br><br>Time since injury: 14.2 years (SD: 11.0).	To compare cardiorespiratory demand and perceived exertion in wheelchair propulsion with and without the use of a mobility assistance dog.	Participants propelled themselves along a 630m outdoor natural environment course.	Quantitative, quasi-experimental design	Oxygen consumption, ventilation, tidal volume, respiratory rate, respiratory quotient, heart rate, and perceived exertion (modified Borg scale).
Chang et al. (2018)	Taiwan, Community sample	n = 30	Men: 70%<br>Women: 30%<br><br>Mean age: 46.8 years (SD: 13.4)	Complete paraplegia: 12<br>Incomplete paraplegia: 1<br>Complete tetraplegia: 12<br>Incomplete tetraplegia: 5<br><br>Time since injury: 20 years (range: 4-41).	To identify environmental factors associated with participation.	Participants speak to the impact of the natural environment on participation.	Qualitative, focus group interview design.	N/A
Chase (2004)	USA, Community sample	n = 1	Women: 1<br><br>Age not reported.	T-12 incomplete paraplegia<br><br>Time since injury not reported.	To describe personal experience of health promotion following SCI.	Participant reflects on her engagement with outdoor experiences while living with SCI.	Qualitative, case-study design	N/A
Chun et al. (2008)	USA, Community sample	n = 15	Men: 66.7%<br>Women: 33.3%<br><br>Mean age not reported. Age range: 27-58 years.	Majority of paraplegia. No further injury-related data were provided.<br><br>Time since injury: 10.7 years.	To explore the characteristics of posttraumatic growth for people with SCI.	Participants described experiences of engaging in outdoor activities.	Qualitative, semi-structured interview design.	N/A
Cooper et al. (2003)	USA, Community sample	n = 4	Men: 100%<br><br>Mean age: 45 years (SD: 2.9).	Injury level: T7 – L1.<br><br>No data on completeness.<br><br>Time since injury not reported.	To observe the IBOT at use in the home and in the community.	Participants used the IBOT to perform a variety of activities, including traversing outdoor surfaces and climbing curbs.	Quantitative, comparative study design	Ability to perform activity and level of exertion.
Curtis et al. (1995)	USA, Community sample	n = 64	Men: 96.9%<br>Women: 3.1%<br><br>Mean age: 42.9 years (range: 23-68).	Cervical: 13<br>Thoracic: 38<br>Lumbar: 6<br><br>No data on completeness.<br><br>Time since injury not reported.	To develop an instrument to detect and monitor shoulder pain for wheelchair users.	Measure of shoulder pain was correlated to various contexts, including outdoors.	Quantitative, cross-sectional scale validation.	Wheelchair User's Shoulder Pain Index (WUSPI)
Daveler et al. (2015)	USA, Community sample	n = 31	Men: 84%<br>Women 16%<br><br>Mean age: 55.8 years (SD: 9.1)	Not reported	To explore conditions and barriers electric powered wheelchair users find difficult to drive in/over in the outdoor environment.	Participants described different barriers to navigating the outdoor environment.	Mixed methods, cross-sectional survey and focus group interviews.	Driving experiences in different driving conditions.

Ding et al. (2008)	USA, Community sample	n = 15	Men: 80%<br>Women: 20%<br><br>Mean age: 38.3 years (SD: 10.5)	Injury level varied from C3 to C7.<br><br>No data on completeness.<br><br>Time since injury: 15.8 years (range: 2-27).	To evaluate the impact of power-assist wheelchairs on mobility, community participation, satisfaction, and psychosocial impact.	Participants reported ease of traversing difficult outdoor terrain and obstacles.	Quantitative, crossover intervention design.	Speed measured with a datalogger.<br><br>Satisfaction with wheelchair (VAS scale). Psychosocial Impact of Assistive Devices Scale (PIADS).
Dorjbal et al. (2020)	Mongolia, Community sample	n = 16	Men: 56.3%<br>Women: 43.7%<br><br>Mean age: 34.9 years (range 25-45).	Paraplegia: 68.7%<br>Tetraplegia: 31.3%<br><br>Complete: 50%<br>Incomplete: 50%<br><br>Time since injury not reported.	To identify environmental barriers and their impacts.	Participants report environmental, seasonal, and meteorological barriers to participation.	Qualitative, semi-structured interview design.	N/A
Dowling et al. (2017)	USA, Community sample	n =10	Men: 100%<br><br>Mean age: 38.6 years (SD: 9.1)	All AIS A-C<br><br>Range of time since injury: 2-20 years.	To explore the feasibility of a device for monitoring pressure relief and physical activity for wheelchair users.	Navigation around a flat 50m outdoor track and a 100m outdoor obstacle course.	Quantitative, feasibility design.	Pressure relief maneuvers and wheel pushes.
Giroux et al. (2020)	Canada, Community sample	n = 22	Men: 55%<br>Women: 45%<br><br>Mean age: 55.6 years (SD: 7.8).	Paraplegia: 64%<br>Tetraplegia: 36%<br><br>Complete: 77%<br>Incomplete 23%<br><br>Time since injury not reported.	To identify barriers and facilitators to participation in daily activities and social roles.	Participants speak to perceived barriers and facilitators to participation, some of which include outdoor activities.	Qualitative, semi-structured interview design.	N/A
Goodridge et al. (2015)	Canada, Community sample	n = 23	Men: 69.6%<br>Women: 30.4%<br><br>Age range: 23-68 years.	Paraplegia: 56.5%<br>Tetraplegia: 43.5%<br><br>No data on completeness.<br><br>Less than 10 years since injury: 47.8%	To examine the perspectives of persons living with SCI on their access to health and support services.	Participants reference the availability of outdoor sporting activities.	Qualitative, semi-structured interview design.	N/A
Goodwin et al. (2009)	Canada, Community sample	n = 4	Men: 50%<br>Women: 50%<br><br>Mean age: 36 years (range: 27-54).	Paraplegia: 50%<br>Tetraplegia: 50%<br><br>All complete.<br><br>Time since injury: 7.5 years (range: 4-15).	To explore the experiences of participating in a group-assisted outdoor hiking excursion.	A weekly summer TrailRider outdoor hiking program over 8 weeks in an urban river valley park. This involved both semi-groomed river valley trails and established groomed trails.	Qualitative, phenomenological case study design with interviews, photographs, and field notes.	N/A

Guillon et al. (2015)	France, Clinical sample	n = 52	Men: 78.8%<br>Women: 21.2%<br><br>Mean age: 38.8 years (range 20-64).	Cervical: 15<br>Thoracic: 29<br>Lumbar: 7<br><br>No data on completeness.<br><br>Time since injury: 12.7 years (range: 1.2-40.5)	To assess differences between manual wheelchairs and 3 pushrim-activated power-assisted wheelchairs.	Participants evaluated each wheelchair on an outdoor course.	Quantitative, single-center, randomized, open-label, repeated measures trial.	Oxygen consumption, heart rate, completion time, push frequency, and satisfaction.
Handrakis et al. (2017)	USA, Community sample	n = 44 (control, n = 41)	Men: 95.5%<br>Women: 4.5%<br><br>Mean age: 48.7 years (SD: 13.8)	AIS A: 16<br>AIS B: 19<br>AIS C: 9<br><br>Time since injury: 17.8 years (SD: 12.1)	To explore differences between SCI and non-SCI regarding the effect of cooler seasons on comfort and performance.	Participants report the effect of cold weather on comfort and participation.	Quantitative, prospective, between-groups design.	Thermal Comfort Survey<br><br>Thermal Activity Survey.
Hartigan et al. (2015)	USA, Clinical sample	n = 16	Men: 81%<br>Women: 19%<br><br>Mean age not reported. Age range: 18-51 years.	Tetraplegia: 3<br>Paraplegia: 13<br><br>No data on completeness.<br><br>Time since injury not reported.	To evaluate mobility outcomes for individuals with SCI after 5 gait-training sessions with a powered exoskeleton.	5-session exoskeleton training period including an objective to walk outside (on concrete walkways and city sidewalks), up and down ramps, and over grass.	Quantitative pilot clinical trial	Gait speed
Haufe et al. (2020)	USA, Community sample	n = 1	51-year-old man	Incomplete C5<br><br>>25 years since injury.	To investigate whether assistance from a wearable robot can increase exercise intensity.	Exercise intensity assessed in an outside walking setting.	Quantitative, case-study design.	Metabolic equivalents
Hitzig et al. (2012)	Canada, Community sample	n = 14 (control, n = 7)	Men: 78.6%<br>Women: 21.4%<br><br>Mean age: 42.3 years (SD: 33.2)	Paraplegia: 50%<br>Tetraplegia: 42.9%<br>Missing: 7.1%<br><br>No data on completeness.<br><br>Time since injury not reported.	To describe the Cottage Program and test its effectiveness.	The Cottage Program is a three-day outdoor experiential therapy intervention, including both green-space and blue-space activities.	Quantitative, pre-post design with non-randomized control group	Goals and satisfaction with program.<br><br>Rosenberg Self-Esteem Scale (RSES)<br><br>Positive and Negative Affect Scale (PANAS)<br><br>Moorong Self-Efficacy Scale (MSES)
Hurd et al. (2009)	USA, Community sample	n = 13	Men: 84.6%<br>Women: 15.4%<br><br>Mean age: 43 years (range: 29-56).	Cervical: 0<br>Thoracic: 10<br>Lumbar: 2<br>Non-SCI: 1<br><br>No data on completeness.<br><br>Time since injury: 17 (range: 1-29).	To quantify manual wheelchair propulsion effort during outdoor community ambulation.	Testing was conducted while subjects propelled their wheelchair over community sidewalk terrain.	Quantitative, case series design.	Propulsion moment, instantaneous power, and work for upper extremities.

Hutchinson et al. (2003)	Study 1: Canada, Clinical sample.<br><br>Study 2: USA, Community sample.	Study 1: n = 6<br><br>Study 2: n = 10	Study 1:<br>Men: 50%<br>Women: 50%<br><br>Mean age: 48.8 years (range: 27-63).<br><br>Study 2:<br>Men: 70%<br>Women: 30%<br><br>Mean age: 36.9% (range: 24-46).	Study 1:<br>Incomplete tetraplegia: 1<br>Complete tetraplegia: 1<br>Other disability: 4<br><br>Time since injury not reported.<br><br>Study 2:<br>Incomplete paraplegia: 1<br>Complete paraplegia: 6<br>Incomplete tetraplegia: 1<br>Complete tetraplegia: 2<br><br>Time since injury not reported.	To examine how individuals used leisure in coping with a traumatic injury or chronic illness.	Participants discussed participation in different outdoor activities.	Qualitative, semi-structured interview design.	N/A
Jean et al. (2021)	Canada, Clinical sample.	n = 159	Men: 79.9%<br>Women: 20.1%<br><br>Mean age: 48 years (SD: 18.42)	AIS A: 31.4%<br>AIS B: 13.8%<br>AIS C: 19.5%<br>AIS D: 35.2%<br><br>Time since injury not reported.	To identify a method for early prediction of independent outdoor functional walking 1 year after SCI.	Prediction of independent outdoor functional walking capacity.	Quantitative, prospective cohort design.	Outdoor functional walking capacity (SCIM).
Kleiber et al. (1995)	USA, Community sample.	n = 20	Men: 75%<br>Women: 25%<br><br>Mean age: 32.2 years (range: 18-59).	Paraplegia: 6<br>Tetraplegia: 13<br>Unclear: 1<br><br>No data on completeness.<br><br>Time since injury not reported.	To offer a conceptual pathway for examining the relevance of leisure in illness experiences.	Interviews reflect on lost or compromised abilities outdoors.	Qualitative, semi-structured interviews design	N/A
Kooijmans et al. (2019)	Netherlands, Community sample	n = 268	Men: 73%<br>Women: 27%<br><br>Mean age: 47.7 years (SD: 8.8).	Paraplegia: 59.3%<br>Tetraplegia: 40.7%<br><br>Complete: 82.8%<br>Incomplete: 17.2%<br><br>Time since injury: 24 years (SD: 9.1).	To examine the relationship between exercise self-efficacy and the amount of physical activity in SCI.	Participants cite various outdoor activities as forms of exercise.	Quantitative, cross-sectional cohort design	Physical Activity Scale for Individuals with Physical Disabilities<br><br>The Spinal Cord Independence Measure III exercise self-efficacy scale.
Koontz et al. (2005)	USA, Community sample	n = 11	Men: 90.9%<br>Women: 9.1%<br><br>Mean age: 50.3 years (SD: 9.7).	Cervical: 1<br>Thoracic: 5<br>Lumbar/Sacral: 3<br>Other disability: 2<br><br>No data on completeness.<br><br>Time since injury: 19.3 years (SD: 11.5).	To conduct a kinetic analysis of manual wheelchair propulsion during start-up on select indoor and outdoor surfaces.	A mobility course consisting of outdoor elements, such as smooth level concrete, grass, and interlocking concrete pavers.	Quantitative, repeated measures comparative design.	Propulsion kinetics.

Labbé et al. (2019)	Canada, Community sample	n = 4	Men: 100%<br>Women: 0%<br><br>Mean age not reported. Age range: 45-70 years.	No data reported on injury level, completeness, or time since injury.	To explore the experiences of an adaptive sailing program and identify factors that impact engagement.	An adaptive recreational open-sea sailing program.	Qualitative design with participant observations and semi-structured interviews.	N/A
Lancini et al. (2019)	Italy, Community sample	n = 1*	Man<br><br>Age not reported.	No data reported on injury level, completeness, or time since injury.	To present a set of instrumented crutches to allow the user-robot interaction to be measured in a more natural setting.	Participant walked 20 steps outdoors on a concrete surface.	Quantitative, case-study study.	Axial forces
Lee et al. (1996)	USA, Community sample	n = 20	Men: 75%<br>Women: 25%<br><br>Mean age: 30.2 years.	Paraplegia: 35%<br>Tetraplegia: 65%<br><br>No data on completeness.<br><br>Time since injury not reported.	To gain insight into how people with SCI perceive their return to previously enjoyable activities.	Participants speak about the continuity of outdoor recreation following SCI.	Qualitative design with participant observations and semi-structured interviews	N/A
Levins et al. (2004)	Canada, Community sample	n = 8	Men: 63%<br>Women: 37%<br><br>Mean age: 42 years.	All thoracic. No data on completeness.<br><br>Time since injury range: 2-27 years.	To explore barriers and facilitators to participation in physical activity.	Participants reflected on barriers and enablers to participation in (occasionally outdoor) physical activity following SCI.	Qualitative, semi-structured interview design.	N/A
Li et al. (2017)	Taiwan, Clinical sample	n = 13	Men: 92%<br>Women: 8%<br><br>Mean age: 42 years (range: 22-57).	Tetraplegia: 66%<br>Paraplegia: 33%<br><br>No data on completeness.<br><br>Time since injury: 1.78 years (range: 0.5-6.2).	To understand the neuropathic pain experienced on their daily life with SCI.	Participants considered how weather contributes to neuropathic pain.	Qualitative, semi-structured interview design.	N/A
Lundström et al. (2014)	Sweden, Community sample	n = 97	Men: 56.7%<br>Women: 43.3%<br><br>Mean age: 43.5 years (SD = 12).	Tetraplegia: 47.4%<br>Paraplegia: 45.4%<br>Not reported: 7.2%<br><br>No data on completeness.<br><br>Time since injury: 7.3 years (SD = 5.6).	To explore leisure repertoire of persons with SCI and relationship to performance, interest, and well-being.	Participants reported on different leisure activities, some of which were outdoors, and interest, performance, and well-being related to these outdoor activities.	Quantitative, cross-sectional cohort design.	NPS-interest checklist
Lysack et al. (2007)	USA, Community sample	n = 136	Men: 62.5%<br>Women: 37.5%<br><br>Mean age: 37 years (range: 18-68).	Paraplegia: 54.4%<br>Tetraplegia: 45.6%<br><br>No data on completeness.<br><br>Time since injury: 11 years (SD: 10.1).	To explore environmental barriers for community integration.	Participants reported the perceived environmental barriers for community integration, some of which were related to outdoors.	Quantitative, cross-sectional cohort design.	Craig Hospital Inventory of Environmental Factors-short form (CHIEF-SF)<br><br>Community Integration Measure (CIM)

Madorsky et al. (1984)	USA, Community sample	n = 1	Man<br><br>Exact age not provided. Based on year of birth and time of expedition, he was 27 or 28 years old.	Paraplegia<br><br>Time since injury: 9 years	To illustrate wheelchair mountaineering as an added modality in the long-term rehabilitation of SCI	Report of a wheelchair-mountaineering expedition of Guadalupe Peak.	Qualitative, case-study design.	N/A
Manns et al. (2019)	Canada, Community sample	n = 11	Men: 63.6%<br>Women: 36.4%<br><br>Mean age: 37.5 years (SD: 13.7)	No data on injury characteristics.<br><br>Time since injury: 7.8 years (SD: 7.9)	To explore the expectations and experiences of adults with SCI training to use the ReWalk exoskeleton.	12 weeks of 4 times weekly training using the ReWalk. Training included stopping, turning, and walking on asphalt and grass.	Qualitative, repeated semi-structured interview design.	N/A
Martin Ginis et al. (2010)	Canada, Community sample	n = 347	Men: 77.8%<br>Women: 22.2%<br><br>Mean age: 45.4 years (SD: 13.8).	AIS A-C: 72%<br>AIS D: 28%<br><br>Time since injury: 13.5 years (SD: 10.0).	To describe the types, intensities, and average duration of leisure time physical activity performed by people with SCI.	Participants recount their recreational outdoor activities and its intensity.	Quantitative, cross-sectional cohort study.	Physical Activity Recall Assessment for People with Spinal Cord Injury (PARA-SCI).
Martin et al. (2017)	Australia, Clinical sample	n = 25	Men: 64%<br>Women: 36%<br><br>Mean age: 44.5 years (range: 19-75).	Paraplegia: 40%<br>Tetraplegia: 60%<br><br>Complete: 40%<br>Incomplete: 60%<br><br>Time since injury in days: 67.2 (range: 17-197).	To identify leisure therapy goals set by individuals with SCI.	Participants expressed therapy goals, some of which were related to outdoors.	Qualitative, longitudinal pre-post design.	N/A
McDaniel et al. (2017)	USA, Community sample	n = 5	Men: 80%<br>Women: 20%<br><br>Age range: 45-59 years.	Range of level of injury: C7-T11<br><br>Range of time since injury: 3-33 years.	To describe Team Cleveland's approach to the 2016 Cyblathlon (i.e., hand-cycling).	Diary entries recount how participants preferred to ride outdoors.	Qualitative, diary design.	N/A
Menzies et al. (2021)	Canada, Community sample	n = 15	Men: 66.7%<br>Women: 33.3%<br><br>Mean age: 48.3 years (range: 25-70).	Paraplegia: 7<br>Tetraplegia: 3<br>SCI - not specified: 2<br>Other disability: 3<br><br>Time since injury not reported.	To explore experiences and impact of participation in outdoor recreation<br>and to identify perceived barriers and facilitators.	Participants reflected on their current experiences with outdoor recreation as well as barriers and facilitators.	Qualitative design using semi-structured interviews and photographs.	N/A
Mukherjee et al. (2000)	India, Community sample	n = 15	Men: 100%<br>Women: 0%<br><br>Mean age: 35.5 years (range: 20-46)	Paraplegia: 8<br>Non-SCI: 7<br><br>No data on completeness.<br><br>Time since injury not reported.	To assess the ambulatory performance of an arm propelled three-wheeled chair device.	Testing of the arm propelled three-wheeled chair took place on a level outdoor track.	Quantitative descriptive design.	Physiological Cost Index

Mukherjee et al. (2004)	India, Community sample	n = 14	Men: 100%<br>Women: 0%<br><br>Mean age: 31.9 years (range: 21-44).	Paraplegia: 11<br>Non-SCI: 3<br><br>No data on completeness.<br><br>Time since injury not reported.	To compare the efficiency of two types of propulsion.	Tested two three-wheeled chairs on an outdoor track.	Quantitative comparative quasi-experimental design.	Propulsion speed, heart rate, oxygen consumption, physiological cost index, energy cost per unit time and per unit distance, oxygen cost and net locomotive energy cost, energy cost per unit of body weight per unit distance traveled.
Mulhollon et al. (2016)	USA, Community sample	Not specified	No data provided.	All participants were SCI. No other data provided.	To describe the adaptive sports programs at a VA medical center.	Description of summer and winter sports facilitated by the Milwaukee VA Medical Center, some of which were outdoors.	Qualitative, descriptive study design.	N/A
Murphy et al. (2022)	USA, Community sample	n = 313	Men: 76.4%<br>Women: 23.6%<br><br>Age groups:<br>18-35 years = 32.9%<br>36-50 years = 33.5%<br>> 50 years = 33.5%	Incomplete paraplegia: 67<br>Complete paraplegia: 79<br>Incomplete tetraplegia: 98<br>Complete tetraplegia: 67<br><br>Percentage injured 3 years or more: 65.2%	To explore associations of residential greenspace and open spaces with psychological well-being among adults living with SCI.	The proportion of greenspace was specified by summing categories supporting vegetation, including forest, shrubland, grasslands, cultivated land, and wetlands.<br><br>The proportion of open spaces was specified by summing land covered by a human-built mixture of constructed material and vegetation, including parks, golf-courses etc.	Quantitative, cross-sectional survey design.	Positive affect and depression (both from the SCI-QOL study's item bank).
Newman (2010)	USA, Community sample	n = 10	Men: 60%<br>Women: 40%<br><br>Mean age: 42.1 years (range: 24-61).	Cervical: 3<br>Thoracic: 7<br><br>No data on completeness.<br><br>Time since injury: 17 years (range: 2-36).	To identify environmental barriers and facilitators to community participation.	Participants prompted to create a photo documentary of one day they were out participating in their community life. Various reflections on the outdoors were made.	Qualitative, photovoice design.	N/A
Noreau et al. (2002)	Canada, Community sample	n = 482	Men: 81.1%<br>Women: 18.9%<br><br>Mean age: 42.4 years (SD = 12.0)	Complete tetraplegia: 24.3%<br>Incomplete tetraplegia: 19.5%<br>Complete paraplegia: 38.0%<br>Incomplete paraplegia: 18.3%<br><br>Time since injury not reported.	To identify the environmental facilitators and barriers to social participation.	Participants respond to a questionnaire wherein several barriers and facilitators were directly related to outdoors.	Quantitative, cross-sectional survey design via phone.	Measure of the Quality of the Environment (MQE)

Oh et al. (2013)	South Korea, Clinical sample	n = 4	Men: 75%<br>Women: 25%<br><br>Mean age: 51.8 years (range: 33-63).	Cervical: 1<br>Thoracic: 2<br>Lumbar: 1<br><br>All incomplete.<br><br>Time since injury: 2.6 years (range: 1.3-3.8).	To describe a community-based ambulation training for ambulatory people with SCI.	The community walk test was performed outdoors near the hospital.	Quantitative, intervention design without control group, pre-post and 1-year follow-up	Outdoor specific: Completion Time (Community Walking Test).
Olmos et al. (2008)	Argentina, Community sample	n = 18	Men: 66.7%<br>Women: 33.3%<br><br>Mean age: 42.8 years (range: 19-72).	Cervical: 12<br>Thoracic: 4<br>Lumbar: 2<br>All AIS D.<br><br>Time since injury not reported.	To determine if a community environment would impact gait performance.	Participants walked outside in the local community during testing.	Quantitative, cross-sectional cohort design.	Gait speed (10MWT and 6MWT).
Pentland et al. (1991)	Australia, Community sample	n = 11	Men: 0%<br>Women: 100%<br><br>Mean age: 42.9 years (SD: 9.6).	All thoracic.<br>All paraplegia.<br><br>Time since injury: 15.2 years (SD: 4.3).	To explore upper limb function and pain in women with SCI using a wheelchair compared to a matched group.	Participants reported the likelihood of experiencing upper-extremity pain from outdoor wheeling.	Mixed methods, Cross-sectional survey and interview design.	Pain (NRS).
Pentland et al. (1994)	Australia, Community sample	n = 52	Men: 100%<br>Women: 0%<br><br>Mean age: 44.3 years (SD: 12).	All thoracic.<br>All paraplegia.<br><br>Time since injury: 17.4 years (SD: 11).	To explore upper limb function and pain in men with SCI using a wheelchair compared to a matched group.	Participants reported tasks contributing to upper limb pain, including outdoor wheeling	Mixed methods, Cross-sectional survey and interview design.	Pain (NRS).
Perrier et al. (2013)	Canada, Clinical sample	n = 14	Men: 71.4%<br>Women: 28.6%<br><br>Mean age: 40 years (range: 21-65).	Paraplegia: 85.7%<br>Tetraplegia: 14.3%<br>Complete: 57.1%<br>Incomplete: 42.9%<br><br>Time since injury: 3.5 years (range: 1-7).	To explore narrative environments in SCI and how these motivative leisure activities.	Participants were to reflect on their motivation for engaging in leisure activities, some of which were outdoors.	Qualitative, Life history interviews.	N/A
Petrofsky et al. (1983)	USA, Community sample	n = 2	No sample characteristics provided.	Both had T10 injuries.	To design and test a three-wheel bicycle for people with SCI.	Bicycle was pedaled outside across a university campus.	Quantitative, case study design.	Fatigue
Ponti et al. (2020)	Italy, Clinical and community sample.	n = 15	Men: 73%<br>Women: 27%<br><br>Mean age: 36 years (SD: 13.4).	Cervical: 5<br>Thoracic: 8<br>Lumbar: 2<br><br>Time since injury: 13 years (SD: 8.6).	To explore satisfaction, quality of life, and fear of falling in sit-skiing.	Participants reported levels of satisfaction, quality of life, and fear of falling associated with prior use of a sit-ski.	Quantitative, cross-sectional survey.	The Quebec User Evaluation of Satisfaction with Assistive Technology (QUEST 2.0)<br><br>World Health Organization Quality of Life (WHOQoL-BREF)<br><br>Spinal Cord Injury Falls Concern Scale (SCI-FCS).

Pradon et al. (2021)	France, Community sample	n = 22	Men: 77.3%<br>Women: 22.7%<br><br>Mean age 47 years (SD: 11 years).	Injuries ranging from C7 to cauda equina. No further data provided.	To explore the effect of power-assistance on upper limb biomechanical and physiological variables during wheelchair propulsion.	Wheelchair propulsion test over a straight outdoor course.	Quantitative, randomized, cross-over design.	Oxygen consumption, heart rate, tidal volume, minute volume, and metabolic equivalent of task (MET).
Rauch et al. (2010)	Switzerland / Thailand Clinical sample	n = 2	Participant 1: Man; 25 years old.<br><br>Participant 2: Man; 24 years old	Participant 1: AIS grade A; T7 paraplegia; 4 months post-injury.<br><br>Participant 2: AIS grade A; C6 tetraplegia; 16 months post-injury.	To explore participation using ICF as a framework and two SCI cases as examples.	Participants reported accessibility issues related to the outdoor environment.	Mixed methods, disability evaluation and outcome assessment case study.	ICF Categorical Profile
Ray et al. (1984)	UK, Community sample	n = 22	Men: 50%<br>Women: 50%<br><br>Mean age: 31.9 years (men) and 32 years (women).	All paraplegia.<br><br>No data on completeness.<br><br>Years since injury: 12.2 year (men) and 9.5 years (women).	To explore the experiences of social, sexual, and personal domains of SCI.	Participants reported appreciating nature more keenly post injury.	Qualitative, interview design.	N/A
Rice et al. (2019)	USA, Community sample	n = 20	Men: 55%<br>Women: 45%<br><br>Mean age: 47 years (range: 19-67).	SCI: 11<br>Other disability: 9<br><br>No data on injury level or completeness.<br><br>Time since onset of disability: 22.1 years.	To examine fall circumstances, recovery process, and influence on community participation.	Manual wheelchair users reported common fall circumstances outdoors.	Mixed methods, cross-sectional survey and semi-structured interviews.	Self-reported number of falls, fear of falling, and activity curtailment.<br><br>Community Participation Indicators (CPI).
Ripat et al. (2012)	Canada, Community sample	n = 19	Men: 68.4%<br>Women: 31.6%<br><br>Age groups:<br>20-39 years = 36.8%<br>40-59 years = 57.9%<br>> 60 years = 5.3%	Tetraplegia: 8<br>Paraplegia: 11<br><br>Incomplete: 4<br>Complete: 15<br><br>Time since injury not reported.	To develop a theoretical understanding of the influences on self-perceived participation for individuals with SCI.	Participants reflected on accessibility issues outdoors.	Qualitative, semi-structured interviews and photovoice design.	N/A
Ripat et al. (2018)	Canada, Community sample	n = 1*	Man in his mid-30s.	No injury characteristics provided.	To explore the patterns of community participation across a one-year period.	Participant reports how winter conditions affect ease, choices, and options of participation.	Qualitative, longitudinal, case study	N/A

Rogers et al. (1978)	USA, Community sample	n = 35	Men: 85.7%<br>Women: 14.3%<br><br>Median age: 24 years (range: 17-52).	All cervical injuries.<br><br>Range of years since injury: 1.5-4.5 years.	To examine the impact of tetraplegia on a person's avocational pursuits in terms of type, frequency, and enjoyment of activities.	Participants rated degree of participation and enjoyment in outdoor activities, now and prior to injury.	Quantitative, cross-sectional descriptive survey.	Leisure inventory
Rojhani et al. (2016)	USA, Community sample	n = 1	Man, 27 years old	Injury: C4, AIS A<br><br>Time since injury: NA	To demonstrate the beneficial effects of adaptive sailing as part of the rehabilitation of an individual with a chronic, high-level SCI.	Blue space: participation in an adapted sailing program.	Qualitative, phenomenological case study.	N/A
Sable et al. (2004)	USA, Community sample	n = 12	Men: 75%<br>Women: 25%<br><br>Mean age: 37.9 years (range: 25-52).	Cervical: 2<br>Thoracic: 10<br><br>No data on completeness.<br><br>Time since injury: 1.6 years.	To explore the experiences transitioning to their home communities and participation in a recreation-based community health promotion program.	Participation in a health promoting program in which experiences of outdoor activities were specifically mentioned.	Qualitative, semi-structured interviews and case study notes.	N/A
Sale et al. (2018)	Italy, Community sample	n = 8	Men: 75%<br>Women: 25%<br><br>Mean age: 43.3 years (range: 21-67).	AIS A: 3<br>AIS B: 4<br>AIS C: 1<br><br>Time since injury not reported.	To investigate the changes in gait pattern undergoing training with an exoskeletal device.	Tests performed outdoors using an exoskeleton over 20 sessions spanning 5/6 weeks.	Quantitative, prospective quasi-experimental, pre-post-design	6MWT,<br>Perceived exertion (Borg Scale)<br>Timed Up-and-Go (TUG),<br>VAS for pain and fatigue,<br>Participant Satisfaction Questionnaire,<br>Spatiotemporal and kinematic parameters.
Shea et al. (2021)	Canada, Community sample	n = 22	Men: 77.3%<br>Women: 22.7%<br><br>Mean age: 42.5 years (range: 22-57)	All tetraplegia.<br><br>Complete: 77.3%<br>Incomplete: 22.7%<br><br>Time since injury: 19.3 years (range: 1-43).	To examine relationships between exercise intensity and different physiological measures during different modes of exercise.	Hand-cycling outdoors on a 400 m track or on the University Campus streets.	Quantitative, descriptive study	Heart rate,<br>Perceived exertion (Borg scale).
Shirado et al. (1995)	Japan, Community sample	n = 108	Men: 90.7%<br>Women: 9.3%<br><br>Mean age: 53.3 years (range: 30-79).	Cervical: 23<br>Thoracic: 47<br>Lumbar: 38<br><br>Time since injury not reported.	To explore how people with SCI performed outdoor activities during the winter season and to determine any special outdoor mobility problems.	Participants described the risks, demands, and necessities of performing winter outdoor activities.	Quantitative, cross-sectional survey design.	Frequency, purpose, mobility method, medical problems, risks, and requirements of outdoor activities (self-made questionnaire).

Singh et al. (2020)	Canada, Community sample	n = 12	Men: 33%<br>Women: 67%<br><br>Mean age not reported but same sample as Singh et al. (2021) below.	Cervical: 4<br>Thoracic: 7<br>Lumbar: 1<br><br>AIS A: 2<br>AIS B: 5<br>AIS C: 4<br>AID D: 1<br><br>Mean time since injury not reported but same sample as Singh et al. (2021) below.	To explore the factors that influenced the risk of falling as perceived by wheelchair users with SCI.	Participants described places or things that they perceived increased and decreased their risk of falling. This included outdoor objects and spaces.	Qualitative, photo-elicitation interview design.	N/A
Singh et al. (2021)	Canada, Community sample	n = 12	Men: 33%<br>Women: 67%<br><br>Mean age: 41.8 years (SD: 12.5 years).	Cervical: 4<br>Thoracic: 7<br>Lumbar: 1<br><br>AIS A: 2<br>AIS B: 5<br>AIS C: 4<br>AID D: 1<br><br>Median time since injury: 17.5 years (range: 3–44).	To explore the psychosocial impacts of falls and risk of falling from the perspectives of wheelchair users with spinal cord injury.	Participants described the psychosocial impacts of falls, some of which occurred outdoors.	Qualitative, photo-elicitation interview design.	N/A
Summers et al. (1988)	UK, Community sample	n = 20	Men: 100%<br>Women: 0%<br><br>Mean age: 28 years (range: 20-39)	Between C8 and T12.<br>All complete injuries.<br><br>Time since injury: 3.42 years (range: 0.25–8.25).	To determine the efficacy of a novel hip guidance orthosis for achieving independent outdoor mobility in SCI.	Community use of the ParaWalker for a minimum of 6 months.	Qualitative, descriptive design with interviews.	N/A
Sung et al. (2019)	USA, Community sample	n = 41	Men: 31.7%<br>Women: 68.3%<br><br>Mean age: 45.5 years (SD: 16.4).	No data on injury characteristics.<br><br>Time since injury: 21.5 years (SD: 17.7).	To explore and compare circumstances of falls among wheelchair users with SCI and multiple sclerosis (MS).	Participants described factors related to falls, of which some occurred outdoors.	Mixed methods, cross-sectional cohort design using survey with both closed and open-ended questions.	Spinal Cord Injury-Fall Concerns Scale (SCI-FCS)
Taylor et al. (1996)	USA, Community sample	n = 3	Men: 66.7%<br>Women: 33.3%<br><br>Mean age: 30 years (range: 23-38).	All incomplete tetraplegia.<br><br>Time since injury: 5.3 years (range: 3-10).	To identify meaningful components of the experience of sea kayaking as described by persons with SCI.	Participants described their experiences of sea kayaking expeditions provided by an outdoor experience organization.	Qualitative, ethnographic design with interviews.	N/A
Tefertiller et al. (2018)	USA, Community sample	n = 32	Men: 84.4%<br>Women: 15.6%<br><br>Mean age: 37 years (range: 18-64).	Between T4 and L2.<br>AIS A: 21<br>AIS B: 5<br>AIS C: 6<br><br>Time since injury not reported.	To assess safety and mobility outcomes utilizing the Indego powered exoskeleton in indoor and outdoor walking.	8-week training protocol consisting of walking training 3 times per week utilizing the Indego powered exoskeleton in indoor and outdoor conditions.	Quantitative, multicenter prospective observational cohort study	10MWT, 6MWT, Timed Up & Go (TUG), and 600-meter walk test.

van Dijsseldonk et al. (2020)	Netherlands, Community sample	n = 14	Men: 50%<br>Women: 50%<br><br>Median age: 29 (range: 24-49)	All thoracic injuries.<br><br>AIS A: 13<br>AIS B: 1<br><br>Time since injury: 6.25 years (0.75-27).	To assess the amount, purpose, and location of exoskeleton use in the home and community environment by people with SCI.	Exoskeleton home and community environment.	Quantitative, repeated measures design.	Steps, usage, sessions, health-related effects (logbook).<br><br>Satisfaction (D-QUEST)<br><br>Usability (System Usability Scale).
van Silfhout et al. (2017)	Cross-European Clinical samples	n = 920	Men: 77.9%<br>Women: 22.1%<br><br>Mean age not reported.	C1-C8, AIS A, B, C: 1.2%<br>T1-S5, AIS A, B, C: 8.5%<br>AIS D: 86.5%<br>AIS E: 3.8%<br><br>Time since injury not reported.	To determine at which walking speed people with SCI tend to walk in the community instead of using a wheelchair and explore clinical conditions that predict independent ambulation in the community.	Outdoor mobility assessed by the Spinal Cord Independence Measure (SCIM).	Quantitative, Retrospective cohort study	10MWT<br>Spinal Cord Independence Measure (SCIM)
Worobey et al. (2022)	USA Clinical samples	n = 533	Men: 76%<br>Women: 24%<br><br>Median age: 46 years (IQR: 34-57.5).	Paraplegia: 51%<br>Tetraplegia: 49%<br><br>Median time since injury: 13 years (IQR: 5-24).	To investigate the frequency and consequences of wheelchair repairs.	Report of those who were active outside of their home, traveling community distances, or encountering outdoor terrain in a wheelchair.	Quantitative, cross-sectional survey design.	Cost and incidence of wheelchair repairs and consequences and wheelchair usage within the past 6 months.

Notes. *Author(s) have confirmed SCI sample or more than 50% of sample were SCI.
SD = standard deviation, IQR = Interquatile range, SCI = spinal cord injury, AIS = ASIA Impairment Scale, 6MWT = six-minute walk test, 10WMT = 10-meter walk test, NRS = Numeric Analogue Scale, VAS = Visual Analogue Scale, ICF = International Classification of Functioning, Disability, and Health

Supplementary Information

Appendix 3. Main findings of the included studies.

Reference	Main findings
Alsaleem et al. (2019)	All participants succeeded in using Tetra-Ski to complete multiple runs on the ski-slopes.<br><br>Participants reported the same or lower anxiety from pre- to post-skiing with the Tetra-Ski. They also reported the same or increased feelings of preparedness to use the Tetra-Ski.<br><br>All participants reported a positive impact on the three subscales (competence, adaptability, and self-esteem) after using Tetra-Ski.<br><br>In the qualitative data, participants expressed a lot of positives experiences and excitement such as: “It's awesome” and “I would not change anything about this experience.” They also reported feeling empowered. For instance, one participant said: “I would be interested in using the ski chair again because it gave me a sense of empowerment over my quadriplegia and control over my environment.” Another described enjoying a sense of autonomy: “I like seeing beautiful things and going kind of fast … [it was] fun being able to choose where I wanted to go.”<br>Multiple participants expressed that the shared control system, where the trainer could take control of Tetra-Ski, made them feel safe, because dangerous situations could be avoided. For instance, one participant said: “The [trainer] helped [me] not get in a lot of wrecks.”
Balbale et al. (2017)	Many participants described participating in outdoor activities. Quote: “Well, there's fishing [and] I got a crossbow... Anything that has to do with the outdoors, I'm there.”<br><br>Another explained the positive effects of using a hand cycle bike to get outdoors. Quote: “That’s important just to live, but this gets me out of the house, through the neighborhoods. As long as there's a paved bike path, I can get up into the woods and ride around and it makes me very happy.”<br><br>Another commented on the positive effects of being outside. Quote: “You’ve got to exercise and it gets me away from all the civilization. I'm a military guy, I like being in the woods. Not here with planes and cars... I love being outside... I really enjoy the adrenaline”<br><br>One person described being happy that he was convinced to go to a winter ski clinic: “I’ve been to Colorado a few times through the hospital. They have a winter ski clinic that was awesome. I didn’t really want to do it the first time, I hadn’t been hurt six months or nine months and they pushed me to do it. I’m so glad I went. I learned so much from all the guys.”<br><br>The cost of adaptive equipment was a barrier to participating in outdoor activities. Quote: “I’d like to hunt and fish. Adaptive equipment related to that to get me to the field again... [is] out there, but they are very costly.”<br><br>Open spaces and all-terrain wheelchairs facilitated participation in outdoor activities. Quote: “I took these pictures because there is a lot of wide open space. I don’t like being in a congested area, this allows me to get out and around” and “I just got a new [action-track] chair. It’s got the tank tracks on it, which allows me to go even more into the woods where a regular wheelchair can't go. This will allow me to go other places so that I can do better fishing or fishing in more places. I can go hunting”.
Barbin et al. (2008)	The skiing program resulted in significant difference for time for global self-esteem, physical self-worth, physical condition, sport competence, and attractive body, while physical strength was non-significant.

	Exploring differences between means pre-skiing and post-skiing as well as between pre-skiing and during-skiing showed that all six of the above-mentioned dimensions were significantly increased from pre-skiing to post-skiing, while only global self-esteem, physical self-worth, sport competence, and attractive body were increased during-skiing compared to pre-skiing.
Barclay et al. (2016)	Of the 13 identified themes about facilitators and barriers to social and community participation, one theme 'environments are inaccessible' were relevant to this review. Almost all participants had experiences of being prevented from participating in activities due to inaccessible natural or built environments. One participant noted: "Here's a new estate that's just been built. If I go into that estate, I have to stay on the bitumen road, because the gutters are too steep, I can't get up the gutters, and there is no footpath, it's just grass", while another stated: "Even the park across the road I either go on the grass which is wet and boggy and whatever or I walk [wheel] around the park basically on the concrete around the park so I'm not enjoying the park."
Beekman et al. (1999)	Participants propelled a standard or ultralight wheelchair around an outdoor track.<br>Using the ultralight wheelchair, participants with tetraplegia and participants with paraplegia propelled further and at greater speeds compared to the standard wheelchair. Oxygen consumption (VO_2) was less only for participants with paraplegia.
Berliner et al. (2021)	At one-year post-injury, 43 participants reported being able to walk one street block outside, while 86 were not.<br>Three MRI indices (i.e., midsagittal ventral tissue bridges, hyperintensity length, BASIC scores) was able to predict outdoor walking ability one-year after spinal cord injury. They did not add additional predictive value over lower extremity motor scores.
Block et al. (2005)	Most of the participants had not participated in the outdoor activities (i.e., kayaking, sailing, fishing, kite-flying, and hand-cycling) since the onset of their disability and did not realize that these activities were possible for them.<br><br>The study reported that during the sea kayaking session, several participants expressed pleasure in learning that they could still kayak. Another participant was thrilled with the kite-flying session, because it was a fun, low-cost activity that could easily be pursued with her children. Another popular session was hand-cycling, because many participants were used to riding bicycles before their disability and did not know that they could still pursue this activity. Some of the participants thought it looked too difficult or were nervous about transferring from wheelchair to hand-cycle but, with encouragement, all participants eventually tried hand-cycling, and expressed that they found it enjoyable.
Boschen et al. (2005)	Winter conditions was greatest barrier to accomplishment of daily life activities for the SCI sample. In total, 84% of the SCI sample reported winter conditions as a barrier. To put this in context, 62% and 60% for support providers and non-SCI members of the general population, respectively, also reported winter conditions as a barrier.
Botticello et al. (2014)	Only the study findings on relationship between open space and participation were deemed relevant in this review, so the associations with residential density, land use mix, and destinations are not reported here. The proportion of open space was specified by summing all the natural land use and land cover including undeveloped forest and wetlands, cultivated farmland, and beach or waterfront (i.e., ocean, lake, or river). The study found that a large proportion of open space (vs. small) in the community (i.e., five-mile buffer) approximately doubled the odds of reporting full participation (vs. restricted) in all domains of participation (physical independence [OR = 2.30), mobility [OR = 1.94], occupation [OR = 1.79], and social integration [OR = 2.32]). Only physical independence remained significant after controlling for social-economic status, demographics and impairment variables (OR = 2.03). A large proportion of open space (vs. small) in the neighborhood (i.e., half-mile buffer) also more than doubled the odds of reporting full participation (vs. restricted) in three of four domains (physical independence [OR = 2.12], occupation [OR = 1.73], and social integration [OR = 2.57]). All these odds were still significant in the fully adjusted models where social-economic status, demographics and impairment variables were controlled for (OR = 2.32, OR = 2.10, and OR = 2.13, respectively).
Botticello et al. (2015)	Only the study findings on relationship between open space and poor health were deemed relevant in this review, so the association with land use mix is not reported here. The proportion of open space was specified by summing all the natural land use and land cover including undeveloped forest and wetlands, cultivated farmland, and beach or waterfront (i.e., ocean, lake, or river). The study found that a large proportion of open space (vs. small) was associated with

	close to half the odds of reporting a poor health (OR = 0.54), but this was not statistically significant in a model controlling for demographics, impairment variables, and quality of life.
Botticello et al. (2019)	Only the study findings on relationship between park space and functioning were deemed relevant in this review, so the associations with residential density, land use, and destinations are not reported here. At the community scale (five-mile buffer), proportion of park space was associated with all domains of higher functioning for people with tetraplegia. For people with paraplegia, significant results were reported for basic mobility, self-care, and fine motor but not wheelchair mobility. At the neighborhood scale (half-mile buffer), having at least one park was associated with higher basic mobility and fewer self-care limitations for people with paraplegia, while none of the results were significant for people with tetraplegia.
Butler et al. (2008)	In validating a physical activity inventory with a subscale 'outdoor work/gardening', this study reported an internal consistency (Cronbach's alpha) of 0.82. They further report a low test-retest reliability for this subscale. In the discussion, the authors state that their participants reported that they rarely performed outdoor activities or gardening, which might have led to the low test-retest reliability.
Caldwell et al. (1994)	In exploring leisure activities, this study reported several comments related to outdoor activities. One person commented on the importance of adaptive equipment after being asked how he felt about this during fishing: "It doesn't bother me because I need it. It's just a fact; I'm injured, I'm not going to be able to do things the same way… but I'm glad that the stuff's there… so I can still do the same things."<br><br>Another noted how outings were an opportunity to practice skills and engage in activities one had engaged in before the injury as well: "I shot rifles before my injury. I was a pretty good marksman, but I never thought about being able to shoot a gun since my injury. I went camping this weekend and was able to shoot a gun."<br><br>It was also noted to just be important for them to get out and away from the hospital setting: "Because being in the hospital for eight weeks, man I'm sick of television, and I am trying a lot of things, going back to fishing. When I have a chance I like to do that. So we tried that last weekend, went to fishing camp. That was a blast. I think the most, the best thing about it was you get out of here. That was the first chance I had to get out of here for a weekend."
Caro et al. (2020)	This study found the 90.9% experienced barriers to their independent mobility on the sidewalk at home and 100% experienced barriers to their independent mobility on the block of their house. The most reported barriers at the sidewalk at home were uneven floor (100%), steep ramps (54,5%), and floors with lateral inclination (36.3%). Earth, sand, gravel and stones as well as holes were considered a barrier by 18.2%. The most reported barriers on the block of the house were uneven floor (81.8%), steep ramps (63.3%), and high curbing (63.6%). Earth, sand, gravel and stones were considered a barrier by 9.1%, while holes were considered a barrier by 45.5%.
Carpenter (1994)	In exploring the experience of SCI, this study found a theme of how to establish a new identity. Within this theme, there was a focus on letting go of the past self to develop a new identity. One quote was related to outdoor activities: "I had to drop out of my two major outdoor activities – backpacking and playing golf – after my injury, but I discovered there are many activities you can get good at. I'm heavily into playing tennis these days, it's a great sport."
Carver et al. (2016)	Over one-third (34.5 %) of all responses mentioned daily obstacles while navigating outdoors, mostly uneven surfaces (e.g., grass, gravel, uneven terrain), curbs (e.g., width and height, lack of curbs), sidewalks (e.g., lack of or unkept sidewalks), ramps (e.g., lack of ramps, too steep), and stairs (e.g., when only stairs are present).<br><br>One in ten participant responses (10.5 %) noted that their mobility assistive device had an impact on their quality of life. For instance, one participant said: ''[I] haven't let it stop me. I am passionate about outdoor living sports, but have continued to do so [in my wheelchair]. I make money doing what I love!''. Another participant noted: ''I've had very few limits for years because of my device; I can do almost anything with my chair – even climb a mountain!'' Others described difficulties with their mobility assistive device: "I cannot go through gravel and outdoors to the places I want to go unless I have my freewheel. I am limited by the accessibility of a place."

Casey et al. (2009)	In exploring the experiences of sea kayaking, this study identified 7 themes, including atmosphere, sense of freedom, sense of achievement, adjustment, equality, safety, and physical benefits.<br><br>Atmosphere: This categorizes the positive experience participants had out on the water. It was described as “enjoyable”, “fun”, “relaxing”, “freedom”, and “fabulous”. Participants who used the single kayak described feelings of flow and being in their own little world. As one person noted: “...I know runners talk about the sort of the flow. But just everything goes perfect and you feel like you could paddle forever... you just get into a stride and you either take off and leave the others behind.” Another expressed: “...if you're a team person or not, you can just do your own thing anyway in kayaking, you can just kind of go at your own pace, generally you'd keep with group but pretty much you can go into your own little world.”<br><br>Sense of freedom: This phrase was used to describe the sense of freedom in mobility that participants felt: “...It’s just the freedom being able to move.... If I'm walking I've stiffness in my legs, I've trouble standing up, my hands don't work.... In the kayak you don’t notice anything like that. You're just like any other person without a disability.” Others used this phrase to describe how it enabled them to explore their environment. As one person said: “... it means that I can go places that I couldn't in a wheelchair. I mean I go into the wilderness of [name of county] up in [name of lake] and go places, and into little inlets ...it's very special. It's the, I suppose it's the sense of freedom ... if I was trying to sell the idea to somebody I would say the sense of freedom.”<br><br>Sense of achievement: Being able to do something new, do it well, and to do it as well as able-bodied gave participants a sense of achievement from kayaking: “...I’d been kayaking before my accident so I kind of had a jist of what it would be like but I didn’t know how I was going to work with being in the chair and getting into the kayak ... and how I was going to manage the oars and stuff like that, but it was grand.... it showed me that there is sport and stuff that I could take part in.”<br><br>Adjustment: Kayaking helped participants come to terms with their loss as it sparked hope that there are still things worthwhile they might be able to do: “When the accident happens and you’re, told like what the situation is you're in a wheelchair and you can't walk and then like your hands won't move, you can't feel....it feels really weird... it (referring to kayaking) made me think; oh I can still do this.”<br><br>Equality: When kayaking, participants felt equal and on the same level as their able-bodied peers as described by the following two quotes: “...you're all kind of on the same playing field so to speak, you're on the same level and everything” and “...because it would happen when our support teams where on the harbours people would be saying “Which is the guy in the wheelchair?” and they would say “I don’t really know!”<br><br>Safety: There were especially two fears present during kayaking; fear of capsizing and the weather, which could be daunting.<br><br>Physical benefits: Participants described several physical benefits from kayaking, including increased strength, endurance, and balance.
Cerny et al. (1980)	The participants either walked or propelled a wheelchair around a 60.5m outdoor cement tract. The study found that, for individuals with a spinal cord injury, walking was significantly more demanding than wheelchair propulsion in terms of energetics, including heart rate, oxygen consumption, and respiratory quotient.
Champagne et al. (2016)	Participants propelled their manual wheelchair around a 630m course in an outdoor natural environment with and without a trained mobility assistance dog. Most participants completed the natural environment course significantly faster with the use of a mobility assistance dog, and further, all cardiorespiratory measures (i.e., oxygen consumption, ventilation, tidal volume, respiratory quotient, respiratory rate, and heart rate) decreased significantly when completing the course with a mobility assistance dog. The participants also reported a lower perceived exertion rate when using a mobility assistance dog.
Chang et al. (2018)	In qualitatively exploring the environmental factors associated with participation, this study found two factors related to the outdoors. The first was ‘accessibility of roads and sidewalks’ where participants expressed the design of these to be a barrier for them. One person said: “The sidewalks are designed terribly in our

	city. Many cross-slopes are too steep for wheelchairs to get on." The second was 'weather' where participants expressed that the weather could sometimes hinder their participation. As one person said: "My power wheelchair cannot be drenched with rain."
Chase (2004)	A reflection on physical activity, health, and wellness combined with the personal story of a person living with a spinal cord injury. This person describes her outdoor experiences with a spinal cord injury and highlights, among other things, the importance of adaptive equipment: "I realize I am very fortunate and grateful to have this variety of equipment as I am able to keep up my active lifestyle and go deep into the wilderness, attain freedom of the road, and work toward a level of optimal physical health that is so important to me." She also emphasizes the importance of getting back into the natural world away: "My interest in exploring the natural world was aided by discovering sea kayaking. This activity I fondly call "the great equalizer" afforded entrance to the backcountry through a water-based activity. I was able to return to the natural world away from the roads, motor vehicles, and the concrete pathways that had become so familiar from the wheelchair."
Chun et al. (2008)	In exploring the experience of post-traumatic growth following SCI, several themes included quotes related to outdoor activities and experiences. In the theme, meaningful engagement, several participants expressed joy and fun in participating in outdoor activities. One person described his first day of participating in a water-skiing clinic, saying "it was a thrill to be able to get back on the water and ski again", while another participant expressed that he loved nature and the outdoors, and that he also liked to visit the park and go into the woods. Another simply enjoyed spending free time outdoors with friends, and that he enjoyed it so much that it outweighed negative physical experiences such as twisted and shaken legs.<br>Others were pleased with the freedom it allowed: "I've gone to the Keys by myself and had a wonderful time. I didn't need to have other people to have a good time. I could just take my tricycle out, and I was riding on those old bridges... I fished".<br>In the theme, newfound appreciation of life, participants expressed a newfound appreciation of beautiful flowers and colourful butterflies.
Cooper et al. (2003)	A number of outdoor activities that were difficult to perform in a manual wheelchair were reported by participants, including climbing steep grassy slopes; propelling through snow or sand; driving through rough and soft terrain; climbing curbs; driving in woods or trails. Participants were able to perform all these activities without assistance with the IBOT (i.e., a dynamically stabilized, advanced wheeled mobility device).
Curtis et al. (1995)	In developing a wheelchair user's shoulder pain index, this study reported a strong and significant correlation ($r = 0.83$) between propelling a wheelchair over uneven surfaces like curbs and grass and shoulder pain.
Daveler et al. (2015)	More than 50 % reported uneven terrain, gravel, driving up steep hills, mud, and wet grass as barriers. Further, more than 50 % specifically avoided mud, soft sand, ice, driving with one wheel off the ground, rain, and cross slopes.<br><br>The most frequently reported obstacles in the interviews where uneven terrain, driving up and down steep hills, cross slopes, gravel, curb cuts, and ramps. The difficulty with these conditions were based on many things, including getting stuck in dirt/mud, slipping and losing traction, driving too fast, motors overheating, and torso being forced to lean to side which is painful.
Ding et al. (2008)	The study found that it was easier for participants to traverse difficult outdoor terrain and obstacles, including ramps, gravel, and grass with a pushrim-activated power-assist wheelchair (PAPAW) than with their regular manual wheelchair. It was also reported that four participants took bike trails in the park with the PAPAW but not with their own manual wheelchair.
Dorjbal et al. (2020)	In exploring the perceived environmental barriers, one theme 'poor access to the physical environment' had one relevant factor labeled 'the outdoor environment'. Here, the participants expressed the inadequacy, or complete lack, of sidewalks and roads with dirt surfaces to be a barrier. As one person said: "Most sidewalks around apartments are not designed and constructed for persons with disabilities" and another mentioned: "Most roads where I live are dirt roads. I mean, we do not have paved roads or sidewalks in rural areas. It is very difficult to get around in a wheelchair because of stones and other obstacles in dirt roads.".

	Participants also mentioned the cold winter weather as a barrier for them. As one person stated: "Well, in summertime, it is much better. I can wait for one or two hours, until someone comes and helps me to get home. But, in winter it is much harder. People with SCI get cold very quickly. When it is –20°C outside, there is a risk getting sick or start freezing. That is why, many wheelchair users do not go outside in wintertime. I stay inside from November to March. In Mongolia, it is –30°C and snowing a lot. The road is very slippery during the winter."
Dowling et al. (2017)	Evaluating a device to quantify physical activity by counting the number of wheel pushes. Participants navigated a flat 50m outdoor track and a 100m outdoor obstacle course that had concrete and grass terrain, inclined and declined ramps, and a curb. The results validated the device.
Giroux et al. (2020)	One participant expressed her need to rely on others to engage in outdoor recreational activities: "Between my husband and my son they can help me get into and out of a lake whether we're going scuba diving, snorkeling, swimming, kayaking, in almost any environment. Because they're strong, they're able to physically help me get through any physical challenges".<br><br>Another expressed the positive benefits he experienced from gardening: "(Gardening) is a big thing lately. It's a thing I took up about three years ago and I'm involved in a community garden. It helps me get a little exercise on the way there, but I also find it very therapeutic in terms of just relaxing".
Goodridge et al. (2015)	These participants expressed their enthusiasm in engaging in a range of outdoor sports, including skiing, cycling, kayaking, and water skiing.<br>However, for participants living further from an urban center, distance was a barrier to engage in these outdoor sporting activities: "It's a long ways to be involved in any kind of . . . team sport with people that are your caliber." Conversely, other participants expressed the benefits of living in a rural area because it is easier to get out and wheel around: "Being in a small town, we don't have near the services like they do in the city, but . . . our preference is to live out here where it's quieter, where you don't have to be driving every place, that you can get out and wheel around and get your exercise."<br><br>A barrier to participation for many was a lack of adequate curb cuts and poor condition of sidewalks. This forced some participants to use the street instead of the sidewalks: "Sometimes we have to go in the streets and off the sidewalk and go to streets and they're just as bad."
Goodwin et al. (2009)	Participants were part of an outdoor hiking excursion program.<br>Thematic analysis revealed four themes: a) off road rush, b) not a wheelbarrow, c) loss of control, and d) interdependence.<br><br>The theme 'off road rush' included the experiences participants had up close with the outdoor trails instead of groomed trails. One participant remarked: "My favorite moment was the very first time I got really up close to the river in the midst of leaves and tree branches and wildflowers and rocks. That very first moment took my breath away. The paved area where people go on runs doesn't get you into the words, which takes away all the beauty of actually being within the environment. I can't wait to go on of the non-paved trails through branches and potholes and getting whipped from tree branches".<br>All participants described the experience with terms such as adrenaline, excitement, and adventure. Especially when going up steep hills and thorugh rough terrain. Several participants also remarked the importance of returning to previous pleasures. Quote: "Ever since the accident I haven't really been by the river, or close to it, so it was a really good experience. Like I didn't want it to end."<br><br>The theme 'not a wheelbarrow' focused on the opportunities that the TrailRider enabled. One participant said: "It's not a wheelbarrow, it's something that gives you the means to get out there and get back to nature." Another said: "I think I've definitively got a positive outlook on the possibilities of getting back into maybe camping, just enjoying the outdoors again."<br><br>The theme 'giving up control' reflected the mixed feelings as the TrailRider meant they had to accept giving up control and being dependent on the volunteers. This was especially pronounced with individuals with paraplegia, for instance: "I had mixed feelings and I guess part of it is I'm quite an independent person. In some ways you almost feel bad that you're asking people to pull you around and I know they're volunteering, and they want to do it. I guess I was a little bit afraid even at the beginning to ask too much of them, or that it would almost be like an imposition on them and yet they do want to be there, and they do want to do it."

	The theme 'interdependence' reflected the feelings of trust and cooperation between the participants and their volunteer helpers. This was an important part of the hiking being an enjoyable experience, and it further provided opportunities for socialization.
Guillon et al. (2015)	Tested three pushrim-activated power-assisted wheelchairs (PAPAWs) against a manual wheelchair on both an indoor and outdoor course. The number of participants who needed help during the outdoor course was 12 with the manual wheelchair and between 8 and 10 with the different PAPAWs
Handrakis et al. (2017)	Asked whether cold seasonal temperatures affected certain aspects of daily living, people with tetraplegia reported feeling comfortable outdoors to a significantly lessor extent than non-SCI peers (17 vs. 43%) and reported having to wear more clothes than others to stay comfortable outdoors to a significantly greater extent compared to non-SCI peers (75 vs. 17%).
Hartigan et al. (2015)	During training sessions with an exoskeleton all participants learned to walk outside, i.e., on concrete walkways and city sidewalks, on ramps with 5-degree slopes, and on grass.
Haufe et al. (2020)	During outside uphill walking sessions, the participant was able to increase his walking speed by 30%, his energy expenditure by 17%, and his heart rate by 9% by using a wearable robot compared to not wearing it. He was able to stay above 0.44 m/s, which was the threshold indicating community ambulation.
Hitzig et al. (2012)	The participants noted that they had engaged in a variety of outdoor activities prior to their SCI (between 2 and 13 activities), while many engaged in very few after their SCI (between 0 and 6 activities).<br><br>The mean perceived number of barriers to participating in outdoor activities decreased during the cottage program (mean_pre = 30.7, SD = 2.1 to mean-post = 16.7, SD = 1.5). This difference was not significant.<br>The frequency of changes in perceived barriers within specific activities were: boating (pretest = 30; posttest = 17), water activities (pretest = 33; posttest = 18), and land activities (pretest = 29; posttest = 15).<br><br>The percentage of participants that noted they would be moderately or very likely to engage in these activities after the cottage program was 17.7% for waterskiing, 29.4% for archery, 29.4% for canoing, 31.3% for kayaking, 31.3% for tubing, 41.1% for fishing, 41.1% for bocce, 47.1% for handcycling, 52.9% for sailing.<br><br>Participants were generally satisfied with the outdoor cottage program with a mean of 4.6 (SD = 1.0) on a scale from 1 to 5.<br><br>Participants in the cottage program experienced significantly increased positive affect and self-efficacy, but no change in self-esteem and negative affect compared to the control group.
Hurd et al. (2009)	In order to quantify manual wheelchair propulsion effort, measurements of moment, work, and power was collected during three different outdoor conditions where participants traversed concrete sidewalks that included smooth level, aggregate level, and a ramp with a smooth surface. Propulsion effort was greater as the resistance increased (smooth vs. aggregated terrain) and as the angle increased from level to inclined.
Hutchinson et al. (2003)	In exploring leisure as a coping resource, several of the themes that emerged reflected outdoor activities for some of the participants.<br><br>In the theme 'leisure as a mental distraction' one participant talked about fishing with his mother: "it's a diversion from worry and stuff like that. It's a lot of fun".

	In the theme 'escaping confines of home and hospital' many participants expressed how the physical environment, particularly their own homes, came to be seen as narrowing and feel like 'solitary' confinement. One participant talked about how getting out was necessary in the process of adjusting to the spinal cord injury; "Going out helped me more than anything because if you sit home you're not going to get adjusted to nothing. If you stay at home all the time when you first get hurt you're just going to waste away."<br><br>In the theme 'providing a sense of belonging and acceptance' one participant described how an outdoor activity like fishing gave him an opportunity to connect with his family: "[Fishing] gives me and my mother an opportunity to do something together that we both like. I don't see a lot of people nowadays really relating to their parents as in things that they can do together and share alike together . . . It's fun, it's fun."<br><br>In the theme 'maintaining physical or mental health' participants talked about how leisure was important to sustain positive mental health, and one quote highlighted the importance of paintball (which was deemed an outdoor activity) for him: "I honestly and truthfully [think that] if I didn't have this [paintball] right now, I don't know what I'd be doing. I'd be nuts." Another talked about the importance of outdoor physical activity for him: "If it's nice outside, I go out and push around because it helps me feel a lot better."
Jean et al. (2021)	The best predictors of outdoor functional walking capacity one year after the spinal cord injury were the highest motor strength for a given myotome and preserved light touch sensation. The sensitivity was 84.2%, specificity was 85.5%, and the overall classification accuracy was 84.9%.
Kleiber et al. (1995)	Hunting and fishing were identified as activities participants used to enjoy doing that they no longer could do or needed modifications to do.<br><br>Participants reported that whilst they could resume outdoor pursuits, they could not engage with the full experience which was highlighted in several quotes:<br><br>"It was great to go back fishing again, but at the same time I can't cast as well, you know; probably one out of five times I got the line in the water, and then one out of fifteen times was actually far enough out to fish"<br><br>"That's really fun to me to sit there and you have to be real quiet waiting on the deer. It's always enjoyment, but it's not the same. You can't get up and move around, I guess. The enjoyment before was that you could get up and you could move around, or you could change places, because you could walk quietly. But now with this wheelchair you've got to sit in one spot, because a wheelchair makes a lot of noise rolling through the woods"<br><br>"I just sit down and fish where I stood before. I haven't been out in the boat yet".<br><br>One participant also expressed how he could not do the same things that he could before but instead engaged in other outdoor activities: "Me and the guys used to get together, and we'd go shoot pool and, uh, which I can't do that anymore. So I just get with the wife, and we just go to the park."<br><br>Others spoke of missed social activities because of outdoor accessibility issues: "like sometimes my group of friends have gone on hikes in the mountains, and I don't go on that because, of course, I can't hike in the mountains. Things like that I miss out on."<br><br>Participants also spoke of a loss of independence: "If I sit down sideways I have to support by one hand or by a back brace which limits me from digging up the ground to plant flowers. I have to have somebody dig it for me, and I tell them I need this planted there" and "Well, sometimes, when I'm at the lake, I have to have somebody help me. I don't like that".
Kooijmans et al. (2019)	In calculating the total physical activity of individuals with spinal cord injury, outdoor wheeling was the domain with the highest median metabolic equivalent value (mean Met 4.8), indicating that, for many respondents, the main form of exercise was wheeling outside. Lawn work/yard care was also one of the 11 domains, but the Met was approx. 10 times lower than wheeling outside (mean Met = 0.5).

Koontz et al. (2005)	Propulsion forces and moments are significantly greater when individuals with an SCI in wheelchairs starting up, propelling uphill, or pushing over grass and interlocking pavements (outside). The study concludes that frequent starting/stopping and propelling outdoor and on inclined surfaces may increase the risk of upper-extremity injuries.
Labbé et al. (2019)	In exploring the perceptions and experiences of an adapted sailing program, two main themes were generated: 1) anchors away: reasons for setting sail and 2) running ashore: challenges with program delivery. In the first theme, many participants expressed the joy of experiencing a combination of freedom and independence out on the water. As one participant with an SCI stated: "I really enjoyed it. At the time I do remember telling people one of the best parts about sailing is it's one of the few activities that I can do without my chair (...) It's a very unique experience compared to other activities, and that was probably one of the most appealing aspects of it." Another reason for sailing was challenging stigmas about their abilities and limitations. As one experienced sailor with an SCI expressed: "I guess taking people out who haven't gone out sailing, you can just tell that they are amazed that you are doing the sailing. So, I guess it change attitudes. It is another great aspect of it." The authors also state that: "Participants described how the immersive nature of this experience fulfilled their desire to engage in a meaningful outdoor activity", but none the quotes given as examples were from individuals with SCI. In terms of challenges, problems with accessibility, scheduling, safety, and reliance on volunteers were mentioned by many, but no specific quotes from individuals with SCI were given. Only one quote was provided by a former sailor with an SCI about why he no longer participated which was due to poor condition of boats due to heavy wear and tear. As he stated: "Over the course of my time sailing, the program became very popular, and the number of boats has pretty much stayed the same, or reduced. There's a high level of maintenance required with their boats. Things were always breaking down, things would get broken."
Lancini et al. (2019)	Tested a novel set of instrumented crutches to assess the load exerted on the upper limbs while using the Rewalk exoskeletal P5 model in an outdoor setting. The study finds that the instrumented crutches could be considered an external validating device for the exoskeleton under natural testing conditions in addition to simulations and experimental gait-lab analysis.
Lee et al. (1996)	In exploring the meaning of continuity in recreation from before to after their injury, several participants talked about outdoor experiences. First, participants expressed how they sought continuity in outdoor activities. One talked about hunting: "Well, I would just go deer hunting, and you usually get two a year or something like that, . . . and I stayed in the woods a lot. I liked the woods and scouted out a lot and that was just a very important part. Like a weekend, I couldn't wait for a weekend to come around and go hunting. I still want to do that. I just have to roll in there now instead of walk." Another talked about going scuba diving: "I guess scuba diving starts next month or the month after training, and I'm going to get put on the list there because I want to learn how to scuba dive. We're going to go down and try it all over again..."<br><br>Some participants viewed continuity as a new hope: "I was just hoping I'll be doing all the same things I learned how to do at rehab. Go fishing, get out and go pushing somewhere, like maybe do a little road race or fun race, and get out and practice doing those. I mean I really don't have the musculature to do it, I really don't have the triceps, like getting up hills is a really hard proposition for me, but I'm hoping that eventually I can go out and do some leisure things and they won't tire me out."<br><br>Even though some of the outdoor activities were performed differently, participants could still find continuity from an experiential perspective. For instance, when participants talked about fishing and hunting: "I went on a hunting outing with them. It was different hunting in a wheelchair, but it was still enjoyable", "I can't cast out as far but I can still cast. . . Basically there's no change. I still enjoy it. Like when I went on the fishing camp back in January; I enjoyed fishing then probably even more than I used to enjoy it before", and "Yeah, just relaxing out in the fresh air, yeah, ... It was basically just to relax because that's what I always used fishing for was just for relaxing, to relax me."
Levins et al. (2004)	In exploring influences on participation in physical activities, two themes emerged: individual and societal.<br>Individual influences included a perceived loss of an able identity: "The outdoor adventure lifestyle was huge to me, and so when I had my accident, it was a major threshold to cross in terms of being able to even see whether that possibility existed or not".

	Several participants expressed that, although experiencing a period of time during which they were sedentary, they eventually reached a point of self-redefinition: “I got really, really involved in riding a bike … I loved it. It felt so good to be pedaling like this, and having a 3-wheel bike, and I was getting noticed by a lot of people. And I just realized that that is something I enjoy doing, I felt my independence coming back.”<br>A few participants described participating in extreme sports, such as skydiving, parasailing, and rock climbing, motivated by the chance to say, “I can do it.” One participant explained how hiking allowed her to reconnect with her family, but also find time to reconnect with herself in solitude: “It was an awesome trip, and you know, I got to sit in a field of daisies, which was one of my dreams, you know [she points to a photo of her sitting in a mountain setting in a field of flowers] and sit by myself. I made everybody else go”.<br>Societal influences included the built environment. One participant recalled having to have others assist her with curbs in her hometown. Another noted an improvement of the built environment with time: ‘’all the curbs have really improved. There wasn’t anything like ramped curbs [back then]”.
Li et al. (2017)	In exploring the experience of neuropathic pain, this study found that, for 11 out of 13 respondents, pain was worsened by cold weather changes. As one person expressed: “The weather is a major factor; our bodies are very sensitive to the weather. We feel more pain in the winter because our nerves are very sensitive.”
Lundström et al. (2014)	Of the 21 leisure activities participants could check, three were performed outdoors: 1) outdoor activities (e.g., walking/wheeling, camping, being in nature), 2) gardening, and 3) fishing, hunting, and shooting.<br>The analysis explored which of these 21 activities were the six most likely and least likely for respondents to be interested in, performed well in, experienced well-being from and experienced changed performance in after their SCI.<br><br>Among the most likely leisure area for respondents to be interested in was the category ‘outdoor activities.’ Conversely, fish/hunting/shooting was among the least likely leisure areas for respondents to be interested in. However, this was not the case for the male sub-sample, who were interested in this area and found it to be a source of well-being.<br><br>In terms of leisure areas participants felt they performed well in, none of the three outdoor activities were among the most likely to be mentioned.<br><br>Among the six most likely leisure areas for participants to endorse as a source of well-being was the category ‘outdoor activities’ for people who had lived with their injury for 2-4 years. Conversely, fish/hunting/shooting was among the least likely for participants to experience well-being from. However, this was not the case in the male sub-sample.<br><br>In terms of experiencing changed performance after the SCI, the category ‘outdoor activities’ was the most likely of all the 21 categories for participants to express changed performance in. Gardening was also among the six most likely categories.
Lysack et al. (2007)	This study found that the greatest perceived environmental barrier to participation was the natural environment (specifically temperature, terrain, climate); more so than government policies, transportation, health services, attitudes at home, help at home, information, surroundings, business policies, prejudice, help at work/school, and attitudes at work/school.
Madorsky et al. (1984)	A case report of one person with an SCI who climbed an 8751-foot mountain in a without wheelchair without any assistance. He encountered many difficulties with parts of the climb being so steep that he had to get out of his wheelchair and pull chair and equipment along by rope clenched between his teeth. At other times, jagged rocks and loose dirt gripped the wheels, so he was forced to maneuver over rocks laid out in front of the chair for traction. After the climb he felt elated emotionally and described it as the most intense emotional experience of his life.
Manns et al. (2019)	Explored the experiences of people with SCI learning to walk with an exoskeleton. Several people expressed positive effects on mental well-being due to being able to walk outside. One person stated: “to be able to go out in the sun and go for a walk – it’s unbelievable what that does for you”, and another one said: “...it was awesome - I kicked a few of the garden leaf piles over, stepped on a few bugs. Haven’t done that in a few years.”

Martin Ginis et al. (2010)	Explored different types of physical activity and how many minutes the previous 3 days people performed these with mild, moderate, and heavy intensity. Most activity types were indoor, but gardening, was deemed of relevance here. Gardening was performed by 8.07 % of the sample, and people performed this activity a mean of 23.15 minutes (mild intensity), 10.77 minutes (moderate intensity), and 7.50 minutes (heavy intensity).
Martin et al. (2017)	In exploring which types of leisure goals individuals with SCI set for rehabilitation, this study found that 26% of their sample set goals within a domain of outdoor hobbies/sports such as “I would like to play wheelchair rugby” and “I want to be able to go fishing again”.
McDaniel et al. (2017)	During cycle training using implanted neural prostheses, this study found that, at first, participants trained indoor at home on a stationary bike, but as they become stronger and less prone to fatigue, they preferred to ride outdoors rather than on the stationary bike.
Menzies et al. (2021)	The most popular outdoor activities mentioned by participants were wheeling, hand-cycling, kayaking, and sit-skiing. More unique activities included scuba diving, horseback riding, and fishing.<br><br>Three themes emerged from the data: a) Into the woods (i.e., current experiences with outdoor recreation), b) ain’t no mountain high enough (i.e., barriers to outdoor recreation), and c) just around the riverbend (i.e., desired changes to outdoor recreation).<br><br>a) Participants closely associated outdoor recreation with recovery and moving forward: “After my injury, I equated outdoor leisure with putting my life back together ... both from a psycho-social perspective and from a physical perspective. For me, outdoor leisure activity was about reintegrating into society.” It also provided a sense of freedom: “And that’s kind of what it was like, we actually entered the forest in ... our bikes in this little path with all the greenery ... it was like the clouds lifted, it’s like this place I could actually traverse on my own and explore some things” and “swimming is also very freeing, you can move around... to be in the water is to be free, right? It’s fun to be able to swim with your grandkids when they only ever see you in a wheelchair ... it lets them see me as someone who can do things.” Participants described encouragement from family and friends as a motivator, and that technology such as adaptive kayaks, paddleboards, and sit skis enabled participation.<br><br>b) There were a range of barriers to participation in outdoor recreation. These included: 1) built & natural environment accessibility, 2) high cost of equipment, 3) transportation, 4) transfers, 5) needing assistance, 6) health concerns such as skin integrity and shoulder overuse, 7) lack of ability to be spontaneous, 8) weather, and 9) the amount of timing or planning an activity took.<br>Negative experiences were also a barrier for some. Specifically, some participants voiced their frustration with the patronizing comments they received from the public, and that these attitudes affected how they participated in on outdoor recreation.<br><br>c) Participants described new adaptations, greater independence, and changes in policy as desired changes. Universal access to adaptive equipment as well as equipment that requires less reliance on other people were sought after. Participants generally found the FreeWheel to be a useful adaption, while the TrailRider was liked the least due to the lack of control, reliance on other, and absence of physical activity. Participants also noted a need to be involved with the city council to provide accessibility (e.g., removing aesthetic features that act as barriers such as patterned sidewalks).
Mukherjee et al. (2000)	Tested an arm propelled three-wheeled chair on an outdoor track. The study found that heart rate can be used to evaluate the performance of the device and to quantify the energy economy.
Mukherjee et al. (2004)	Tested two three-wheeled chairs – one powered by cranking using one arm and the other by using two arms – on an outdoor track. The freely chosen speed was significantly higher and the physiological outcomes were significantly lower for the two-arm chair compared to the one-arm chair. The difference in heart rate and oxygen consumption was not significant.

Mulhollon et al. (2016)	Describes different outdoor activities based on the authors observations and experiences from running a year-round adapted sports program for veterans with an SCI. For summer sports, they describe kayaking as "beneficial for the veterans to be able to escape their homes and the city life. It allows them to get out on a lake for fresh air and provides a means of mobility in the open water." They further describe water-skiing in the same way as it also: "allows the veterans to escape from their daily lives and enjoy the scenery at a lake." Lastly, they describe that hand cycling is: "the most popular adapted sports programs that is offered to the veterans". They have two rides: a 2-4 miles short ride around the campus and an 8-10 miles long ride around a nearby lake. Participants hand cycle outdoors all year around if the weather allows it. For winter sports, only skiing is performed outdoors.
Murphy et al. (2022)	This study explored the association between amount of residential greenspace and psychological well-being.<br><br>The results showed that people living in communities (five-mile buffer) with moderate amounts of natural greenspace experienced significantly less positive affect and greater depression compared to people living in communities with low natural greenspace after controlling for demographic, injury-related, and socioeconomic variables. There was no significant difference in depression or positive affect between the people living in the highest compared to the lowest greenspace communities.<br>At the neighborhood level (half-mile buffer), natural greenspace was not associated with depression nor positive affect.<br><br>In terms of developed open space, there was no effect on positive affect nor depression at the community level. There was however an effect at the neighborhood level in that living in neighborhoods with a moderate amount of developed open space was significantly more depressed and experienced less positive affect compared to people living in neighborhoods with a low amount of developed open space after controlling for demographic, injury-related, and socioeconomic variables. There was no significant difference in depression scores or positive affect between the highest and the lowest neighborhood open space.
Newman (2010)	Using photovoice to explore barriers and facilitators of participation, this study found that getting out of the house was important, but the natural environment was often a barrier. In terms of its importance, one participant expressed: "Life could be like a caged bird if you let it. How would that bird feel? It can't get out of that cage. Every now and again I get that feeling, like I'm caged in ...and, if you caged a bird up long enough, that bird is going to die, see, just from heart trouble, you know. They need to get out from those four box walls, get out to the beach, get out to the shopping center, and get out and smell the roses, you know... these things here make me feel like I'm out of the cage. So, you can kind of see how important it is."<br><br>Factors classified under "natural environment" was mentioned relatively few times and only as a barrier. The most frequent barrier was wind and rain, while some participants also mentioned the challenges of traversing overgrown landscaping that was blocking the more accessible paths.<br><br>A few photos with accompanying quotes from other categories than "natural environment" contained outdoor elements. There was a photo of a parking lot, where the participant described the necessity of having enough space around the car: "This was the only spot I could find. You need an extra 3 foot beyond the end of your ramp on the van, and, and if you don't have...let's say about at least 5 foot space from that yellow line to the curb, you're going to run into a curb, and then that's a problem." Another photo shows a poorly maintained sidewalk and the participant writes: "What I'm showing here in this neighborhood, it's not an affluent neighborhood. You've got a series of really bad sidewalks, no curb cuts, bad curb cuts. But then you go to the more affluent or more populated neighborhood downtown, and they're great." Another used his photographs to: "show people [who] are newly disabled things that they can do they might not realize they can do— like hunt, fish, go on trips." Lastly, there is a photo of a set of stairs outside a bar/restaurant accompanied with the text: "These aren't just any steps. At the top of these steps sits a local bar/restaurant and all around local hangout favored by many of my coworkers. Why am I showing them to you? I mean, I know you've seen steps, and while these are really nice brick steps, they don't appear to be anything special. But they are! These steps provide access to much of the social interaction between my friends at work. These steps hold the insight to private jokes and conversations of people whose company I really enjoy. These steps lead to the way for me to interact with friends. These steps stop me in my tracks. There is no ramp. These steps are my enemy."
Noreau et al. (2002)	In exploring the perceived influence of environmental factors on social participation all the outdoor-related factors were considered mainly obstacles, with winter climatic conditions as the greatest obstacle followed by unevenness of terrain, nature of terrain's surface (grass, sand etc.), and lastly summer climatic conditions.

	Winter climatic conditions: of 443 responses, 53% considered it a major obstacle, 21% a moderate, and 14% a minor, while 3% considered it a major facilitator, 1% a moderate, and 2% a minor.<br><br>Unevenness of terrain: Of 426 responses, 23% considered it a major obstacle, 13% a moderate, and 21% a minor, while 7% considered it a major facilitator, 5% a moderate, and 8% a minor.<br><br>Nature of terrain's surface: Of 419 responses, 18% considered it a major obstacle, 14% a moderate, and 18% a minor, while 7% considered it a major facilitator, 7% a moderate, and 7% a minor.<br><br>Summer climatic conditions: Of 391 responses, 14% considered it a major obstacle, 13% a moderate, and 23% a minor, while 6% considered it a major facilitator, 7% a moderate, and 7% a minor.
Oh et al. (2013)	Tested the effect of a four-week training program on community walking ability measured on an outdoor 300m route that included stepping over obstacles, moving up and down ramps, and crossing the street. The training sessions were sometimes performed indoors (e.g., hallway of hospital, shopping mail) and other times outdoors (e.g., on a sidewalk, in a parking lot, at a cross-walk). The results showed that participants were able to complete the community walking test faster at post-training compared to pre-training (the decrease in time it took ranged from 1.78 min to 15.10 min across participants), but that the gains were mostly not maintained at four-week and 1-year follow-up.
Olmos et al. (2008)	This study found that a community 6min walking test was more representative of the individual's performance as the 6min walking test performed in an indoor gymnasium underestimated the walking ability compared to the same test performed in a natural community setting.
Pentland et al. (1991)	The activity where women with paraplegia experienced the second most pain after 'work/school' was 'outdoor wheeling' with 89% reporting pain from this activity. As pain was rated on a Likert type scale, it was evident that only during 'outdoor wheeling' did the majority (63%) experience pain 'usually' or 'always'.
Pentland et al. (1994)	'Outdoor wheeling' was ranked no. 4 in percentage of men who reported upper limb pain from a range of activities performed during the day. Specifically, 46% reported pain from 'outdoor wheeling'.
Perrier et al. (2013)	In exploring the narrative environments in active rehabilitation, this study found three stories: restitution narratives, chaos narratives, and the quest narrative. The authors report that people who constructed their SCI experiences using the quest narrative were more open to engaging in sports and outdoor recreation if it would provide an opportunity for social connection, enjoyment, and positive experiences with SCI-peers. This was compared to people using the restoration narrative who viewed leisure time physical activity as instrumental for health and was therefore accessed for its functional benefits alone.
Petrofsky et al. (1983)	Described the development of an outdoor bicycle for exercise. Two individuals with paraplegia were able to bike more than 15 minutes outdoors using this bicycle. One downside of the outdoor bike was that people could become fatigued without knowing it, so they had to fit a fatigue meter on the bike.
Ponti et al. (2020)	Sit-skiing was significantly correlated with increases in physical and psychological quality of life, but not social and environmental quality of life. Users were satisfied with the weight, stability, safety, and professional services of the equipment, but not the ease of use and durability. Lastly, fear of falling was not a significant concern for participants.
Pradon et al. (2021)	Tested a power-assistance wheelchair on a straight outdoor course. Participants could propel significantly further with the power-assisted wheelchair compared to a manual wheelchair. Furthermore, peak mechanical effort, heart rate, metabolic equivalent of task, tidal volume, minute volume, oxygen consumption, and peak oxygen consumption were all significantly lower.

Rauch et al. (2010)	In exploring the utility of the ICF to identify problems and needs in participation, this study gives examples from two case studies. One of the participants was completely restricted in outdoor mobility due to a lack of environmental facilitators and barriers in public areas. He therefore had to stay inside all day, and he expressed how the most important thing for him was getting out of the house: “To move inside the house is no problem. But I am inside 24 hours a day. My biggest problem is not being able to get out...not being able to get around outside... I can’t go out, work like work in the field, but I started working with the computer...” The other participant was less restricted, especially inside, but moving around outside still caused moderate problems.<br>While one of the participants was able to meet his friends in public areas due to good wheelchair access, the other was only able to talk to his friends on the phone. In terms of recreation and leisure, both participants experienced restrictions, and even the participant with the fewest restrictions did not participate in any of the outdoor leisure and sport activities that he had engaged in before the injury (i.e., riding motorcycle, playing golf).
Ray et al. (1984)	In exploring the personal, social, and sexual adjustment to an SCI, there were two quotes related to nature experiences. One person said: “Before I was never in (at home). I was going through life with my eyes shut. I wasn't taking any notice. I mean I didn't stay up and watch the dawn come out and listen to the birds and look at the sky and just look at a flower and think how marvelous…”, while another said: “I try now to be more observant. I find myself now deliberately trying to look, to observe nature.”
Rice et al. (2019)	Describes the fall circumstances for people with SCI. There were four themes in their data, and two of these had codes that were related to the outdoors.<br><br>In the ‘time of fall’ theme, poor lighting at nighttime influenced their fall: “I hit something with my front casters, my front wheels and went flying out of my wheelchair and landed in the mud in the middle of the night.”<br><br>In the theme ‘location of fall’, it was found that many falls occurred in the street/sidewalk and driveway. It was further reported than presence of debris often caused their falls: “I was walking my service dog and a rock or twig caught my left caster and I fell out of the chair onto the pavement.”
Ripat et al. (2012)	Public spaces such as parks and playgrounds were generally inaccessible, with ground surfacing identified as the most problematic feature.<br>Sub-zero temperatures, snow and ice were an ongoing challenge. Some participants avoided or minimized outdoor activities, whereas others utilized strategies to address winter barriers, such as driving their wheelchairs on the road to avoid impassable sidewalks: “In the summer I’m a pedestrian and in the winter I’m a car”. Although some barriers might be surmountable, the modifications required made engaging in the activity unacceptable: “I’ve tried every which way to try to get back to golf. I was an avid golfer and I’ve tried different chairs and different ways to hold them it just wasn’t there.”
Ripat et al. (2018)	One of the three participants, Lucas, had a spinal cord injury. He did not let winter conditions (i.e., cold temperatures, snow) stop him from getting out, but it did affect the ease of participation, and he often did a lot of pre-planning (e.g., phoning places ahead of time, checking Google Street View for accessibility) and sometimes changed his plans due to inaccessible environments. He further noted that he found it easier to wheel outside in the snow when it was hard and packed compared to soft or slushy. In sum, the winter conditions did not affect the overall participation, but it affected ease, choices and options for him.
Rogers et al. (1978)	This study found that, following their tetraplegia, 86% experienced decreased participation in outdoor activities (3% experienced an increase) and 34% experienced decreased enjoyment in outdoor activities (3% experienced an increase). Agreement between level of participation and enjoyment was 17% (e.g., increase in participation and increase in enjoyment or vice versa), while disagreement was 83% (e.g., decrease in participation, but increase in enjoyment or vice versa).
Rojhani et al. (2016)	A case-study of one person with an SCI who learned how to sail independently using sip and puff controls. He described that the sea had a generalized calming effect, and he expressed that “being alone was a rush and flipping the boat was a real possibility”. He further highlighted that one of the inherent positive effects of sailing was the “return to nature”. He also stated: “It was great to get out there and compete again because I was out there doing this by myself. That I could get in this boat and just go, and I didn’t have to rely on somebody else, it was just such a great feeling.” After the first sailing event, he described improved mood, self-worth, and community belonging. The responses he gave were solely positive, and the positive effects were noted again a year later at the second sailing event. He also demonstrated improved function following this activity.

Sable et al. (2004)	Explored perspectives on project PATH. One participant talked about the importance of having someone with knowledge about how to pursue outdoor activities to facilitate his involvement in these activities: “Terry had a ton of information on like, all the sports stuff. I remember Terry told me a bunch of different things and was real good on showing me different stuff. And coming up with ideas, you know, how can you do something different. Anything I wanted to know about, you know, like about fishing or being in a boat or anything to do with outdoor, Terry knew about it.”<br>Another mentioned how the availability of equipment to try facilitated his pursuit in outdoor activities: “Darren brought 5 different cycles for me to try which I really appreciated and was amazed. So that was a benefit of PATH. PATH facilitated that. If it wasn’t for path, I would not have been able to at least try kayaking. I probably for a long time would have wondered if I could go kayaking.” The same was highlighted several other participants: “… he [Darren], brought up a bicycle. It was pretty cool. We both took a ride down the street and back. He’s great that way. I have to see how to adapt things, not just hear about it or read about it, and that’s one of the things they (PATH) can offer you. It’s to actually see how I can go ride an ATV or even mowin’ my lawn”, “I never would have gone skiing if I didn’t get into this program or get on a skate and go skating on Sundays” and “PATH allowed me to use the bi-ski … So I would not have been able to go skiing last week if I had not PATH’s bi-ski. I know that there’s a risk you take with that stuff too but, it’s great for me. I was able to go skiing this week because I had the bi-ski. And it’s like no problem. And that’s pretty cool.” Participants further implied that these outdoor activities acting as distraction form their pain: “And too, if you are out skiing I don’t think of it [the pain] as much. So you do things that make you feel better and keep your mind off it. That’s kind of the trick.”
Sale et al. (2018)	Participants trained mobility with an exoskeleton. At post-training (20 sessions) participants were able to walk significantly further in an outdoor 6-minute walk test compared to after 3 sessions. Perceived exertion was not significantly different between the two time points.
Shea et al. (2021)	This study explored whether heart rate or perceived exertion could predict oxygen uptake, and participants exercised in three different modes, one of which was hand-cycling outdoors. Results were inconsistent.
Shirado et al. (1995)	Describes outdoor winter activities. 60.2% had many opportunities to go outside during the winter, 25.9% had few, and 13.9% had no opportunities to go outside. The reasons stated were no outdoor mobility method and too cold weather. The average time spent outside was 3hr and 37min per day and 3.4 days per week. Daily exposure to coldness outside was 30min average.<br><br>Participants who were outside less than 30 min per day were more likely to experience a medical problem compared to people who were outside more than 30 min per day.<br><br>Of the participants who had the opportunity to go outside, 62.4% reported at least one medical problem during outdoor activities. Pain/numbness occurred frequently by 67.2%, upper respiratory infection occurred in 29.3%, spasticity of upper and lower extremities in 17.2%.<br><br>Regarding risks during outdoor activities, 63.4% had experienced potentially hazardous situations. Wheelchair slipping on icy roads resulting in possible collision with a vehicle was reported by 42.4%, tips or falls were very common for 35.5%, and frostbites were reported by 15.3%.
Singh et al. (2020)	In exploring factors that influence the risk of falling, several participants recalled experiences of their wheelchairs tripping over cracks, potholes, drainage grates, rocks, branches, or snow/ice when wheeling outdoors. Participants further described how they were constantly scanning their environment and being continuously alert as a strategy to reduce the risk of falling. Small casters were another issue as they often got stuck in small cracks or caused the wheelchair to become unstable due to debris in the environment.
Singh et al. (2021)	Describing the psychosocial impact of falls through photo-elicitation. Several participants noted how the risk of falling prevented them from engaging in certain recreational activities. One person said that she enjoys wheeling outside for exercise, but the risk of falling has now caused her to stop and instead wheel inside on a track which was less enjoyable to her: “I can’t get the same fast push on when I’m in the neighborhood because I always have to be staring at the ground to make sure I don’t hit anything and go flying...I like going on the track at the gym because there’s no bumps ... but I hate wheeling on the track because I’m in the

	circle and I'm not outside enjoying the fresh air and the nice weather...it does take away some of my ability to exercise the way I want to." Similarly, another participant mentioned that he does not go into his backyard to access his pool because there was a higher risk of falling in this area.<br>Others still engaged in outdoor activities but described several issues. First, they described feeling stressed because they had to continuously scan the environment to find and avoid risk factors, and that this constant scanning detracted from being able to enjoy activities. As one person said: "When I am out for walks with my son in the neighborhood and stuff. I just really have to be diligent like looking at the ground to see if there's anything in my way debris-wise...It just becomes one more thing to worry about...I am a bit more apprehensive. A little bit more nervous." Another person noted that she had to constantly re-adjust her feet on the footplate: "I spent literally half my time when I'm wheeling around the neighborhood either putting my feet back on my footplate because they get bounced off because of, like, the bumps between the sidewalks or just having to pop little wheelies the whole time, so I don't go flying. It's really frustrating because I spent my entire walk staring at the ground looking out for bad sidewalks."
Summers et al. (1988)	Participants were issued a ParaWalker (hip guidance orthosis) for a minimum of 6 months. At follow-up, 8 out of 20 participants had achieved independent outdoor mobility defined as frequently walking more than 50 meters outside over different terrain, including grass, gravel paths, and pavements.
Sung et al. (2019)	In exploring factors related to falls, location emerged as a category. This category included two outdoor locations, garden (6%) and street (22%). In terms of the streets, specifically, several participants mentioned surface conditions on sidewalks: "I was pushing my wheelchair on campus and rolled over a crack in sidewalk" and "I was wheeling down street while intoxicated and distracted. I hit a bump on the sidewalk and I fell" and "I fell down on the street when I was pushing my wheelchair. I rolled over a crack in the sidewalk" and "When I was wheeling down street, I was distracted and ended up hitting a bump on the sidewalk and fell."
Taylor et al. (1996)	In exploring the meaning of sea kayaking after participation in recreational kayaking, this study found seven themes that emerged: 1) atmosphere, 2) social interaction, 3) adjusting, 4) healthy lifestyle, 5) "I can do this", 6) safety, and 7) need for support.<br><br>Atmosphere: Participants described kayaking as a whole other world. One person said: "You get excited about nature, clouds, and the currents." Another described being on the water as relaxing and fun with "laughter that's not forced, but laughter from having done something fun. It's one of the best highs you have." When the authors examined the data closely to find out which factors that constituted this "fun" and "other world" aspect, the aquatic outdoor environment was the most common co-occurrence.<br><br>Social interaction: Participants described friendships forming and socializing where the emphasis was no longer on the injury but on kayaking.<br><br>Adjusting: Sea kayaking was described by participants as a way to defuse the stress of adjusting the inevitable life changes from an SCI. It was something to look forward to when focus was primarily on rehabilitation.<br><br>I can do this: Sea kayaking had a normalizing aspect to it: "I'm just as able as anybody else, when I'm in a sea kayak."<br><br>Healthy life-style: Sea kayaking was also a way to stay healthy both physically and mentally. Participants specifically reported increased strength, stamina, and balance.<br><br>Safety: Participants all felt safe in the kayak: "I really did, I felt safe in a kayak."<br><br>Need for support: All participants expressed a desire for more support for them in pursuing not just kayaking but also other outdoor activities.<br><br>Other perspectives: A general feeling among the participants was how few other activities provide such as equalizing setting: "One of the best things about kayaking may be that it takes the wheelchair out of the activity."

Tefertiller et al. (2018)	Participants completed an 8-week training program with an exoskeleton in indoor and outdoor conditions. During the training participants improved their speed and independence in both indoor and outdoor settings. Indoor and outdoor walking speeds were also not significantly different from each other.
van Dijsseldonk et al. (2020)	Participants used an exoskeleton at home and in the community, and the most common location of use was outdoors (48%). Participants were satisfied with the exoskeleton and its usability. One of the factors related to non-use was weather conditions (e.g., storms, snowstorm).
van Silfhout et al. (2017)	A walking speed of 0.59m/s was able to distinguish between who walks and who does not walk outdoor with a sensitivity of 91.6% and a specificity of 80.3%. Further, age, injury severity, and lower limb muscle strength were also significantly associated with outdoor mobility.
Worobey et al. (2022)	Participants who used their wheelchairs in outdoor terrain (i.e., traversing soft terrain, traversing curbs, and traversing steep inclines) were more likely to experience having to repair their wheelchair that those who used it indoor. They also experienced more consequences as a result, including being stuck in bed, forced to use backup chair, and missed social events.

Notes. SCI = spinal cord injury, OR = odds ratio, PAPAW = pushrim-activated power-assist wheelchair,
